# Supplementary material for: Automating population construction and parallel simulation of biophysical models for neuromuscular cells: An inverse approach
Source: PLoS Comput Biol. 2026 Apr 24;22(4):e1014184. doi: 10.1371/journal.pcbi.1014184 (PMC13132451; doi:10.1371/journal.pcbi.1014184)
Supplement: S1 Text — (PDF) [file pcbi.1014184.s001.pdf]

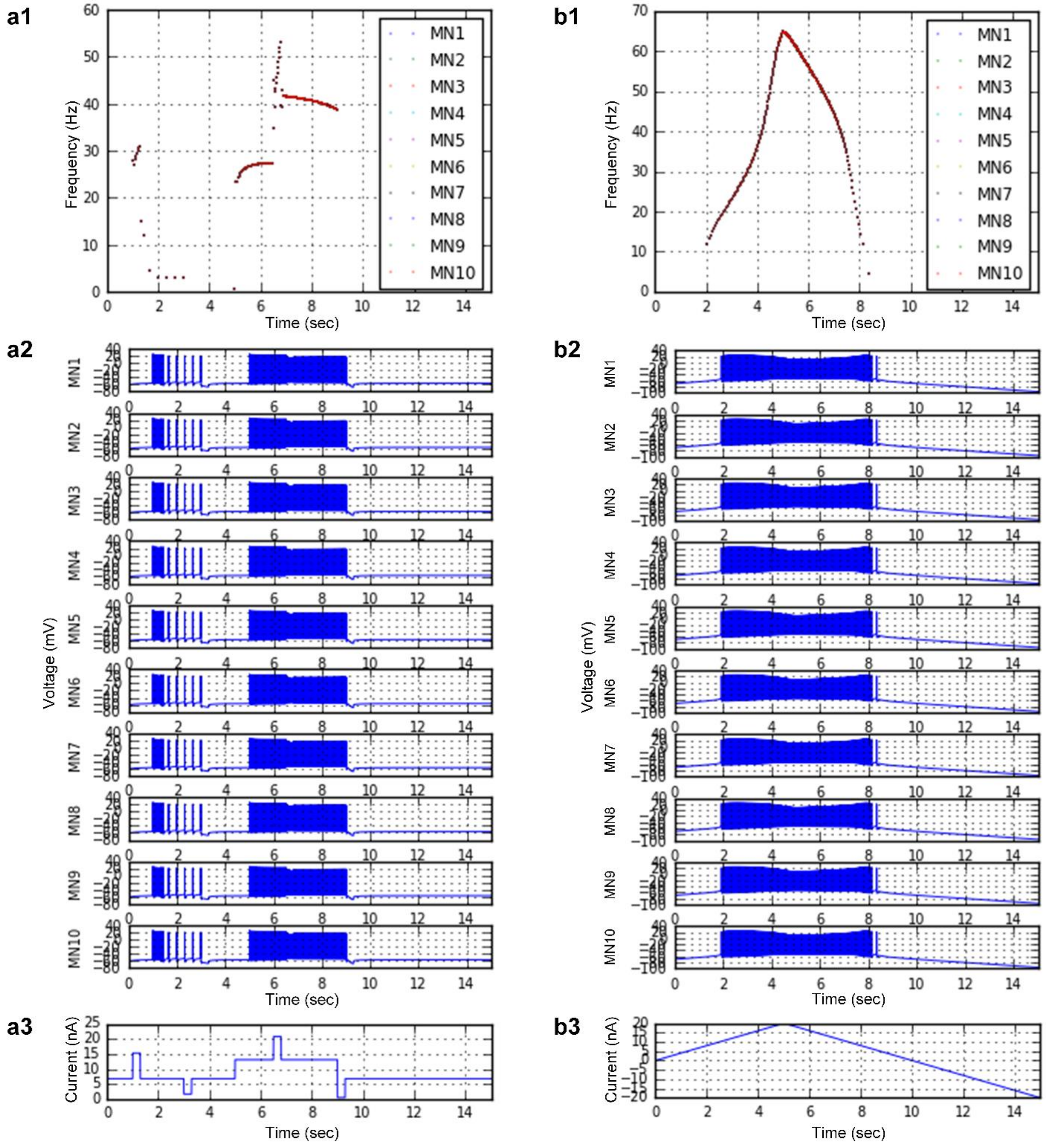

Fig A. Homogenous MN population behavior I. A homogenous population model consisting of 10 FR-type MNs with the same CPs was constructed and simulated as reported in a previous study (1, 2). **a1-a3.** Instantaneous firing rates (**a1**) and transmembrane potentials (**a2**) of individual MNs in response to the alternating step current (**a3**) intracellularly injected at the somata of the MNs. **b1-b3.** Instantaneous firing

rates (**b1**) and transmembrane potentials (**b2**) of individual MNs in response to triangular current injection (**b3**) into the somata of the MNs.

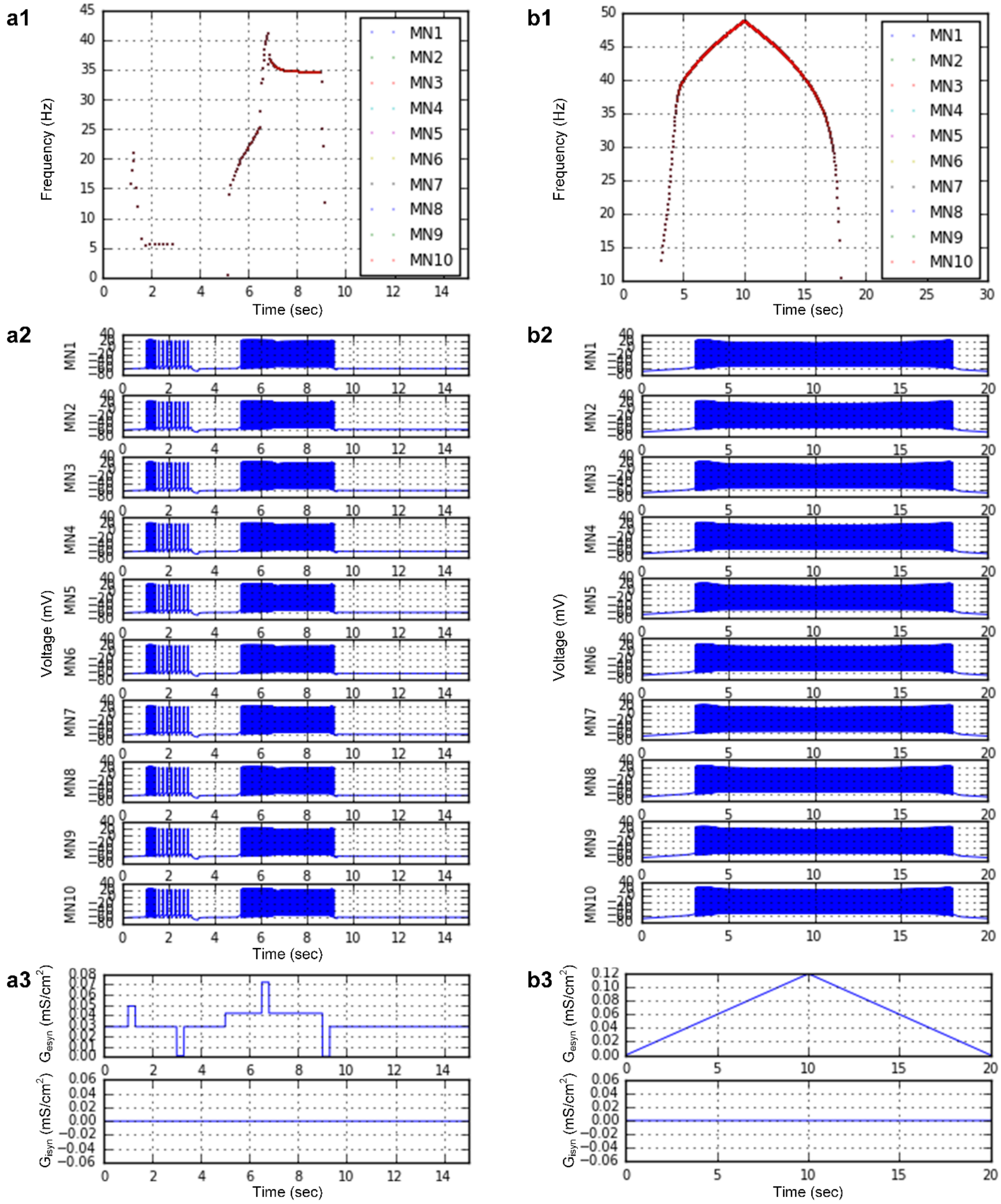

Fig B. Homogenous MN population behavior II. The same population model used in Fig A was simulated as that reported in a previous study (1, 2). **a1-a3.** Instantaneous firing rates (**a1**) and transmembrane potentials (**a2**) of individual MNs in response to the excitatory step synaptic input (**a3**) over the dendrites of the MNs. **b1-b3.** Instantaneous

firing rates (**b1**) and transmembrane potentials (**b2**) of individual MNs in response to the excitatory triangular synaptic input (**b3**) over the dendrites of the MNs.

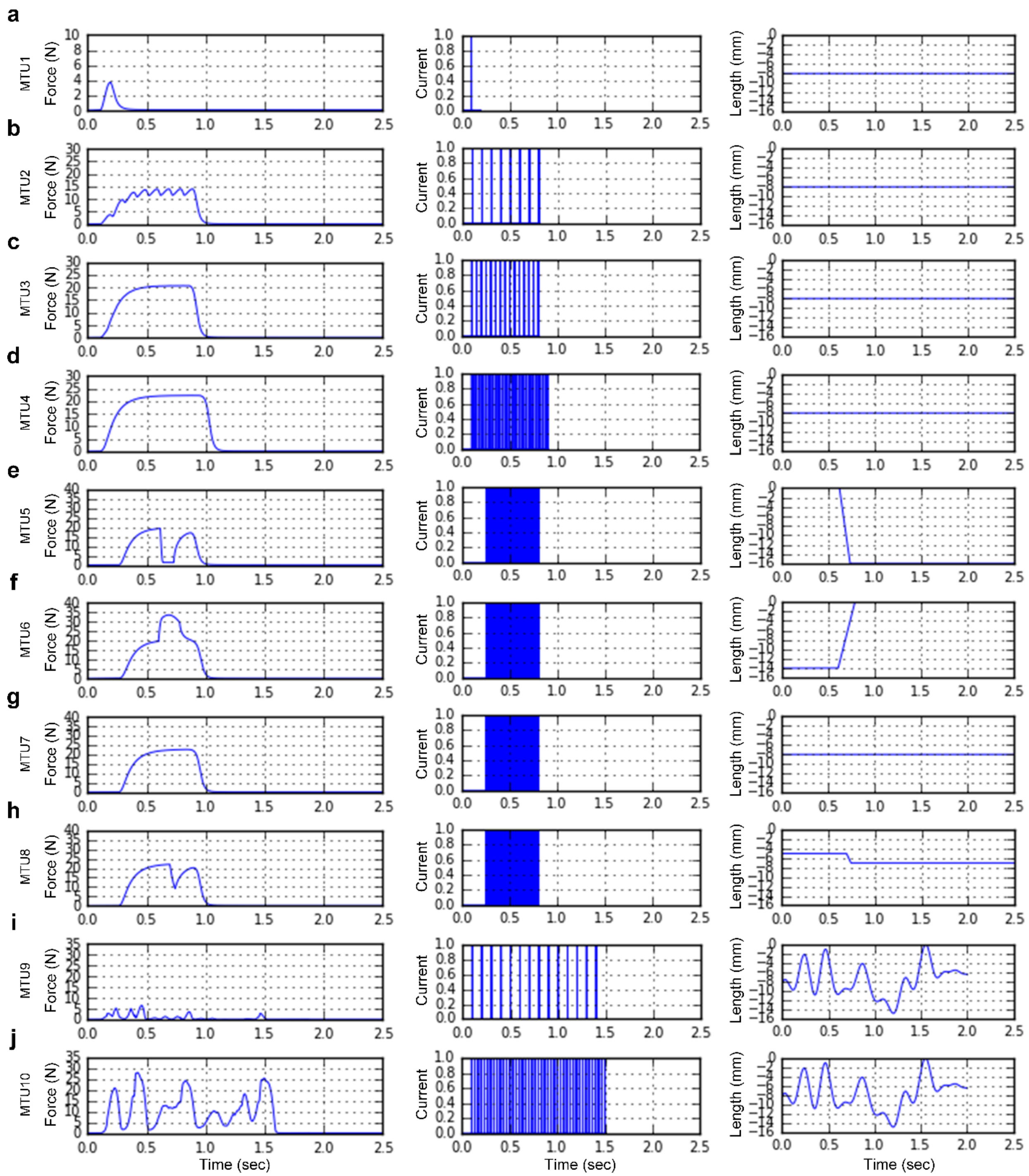

Fig C. Homogeneous slow MT population behavior. A homogenous population model consisting of 10 slow-type MTs with the same CPs was constructed and simulated as reported in a previous study (3). **a-d.** Unfused tetani of individual MTs (left) at constant stimulation frequencies (1, 10, 20 and 40 Hz for **a-d**, respectively) (middle) under isometric conditions at the intermediate length ($X_{m,0.5}$=-8 mm) (right). **e-h.** Fused tetani of individual MTs (left) under full excitation (100 Hz) (middle) during muscle shortening (**e**), lengthening (**f**), isometric (**g**), and step shortening (**h**) (right). **i-j.** Unfused

tetani of individual MTs (left) at constant stimulation frequencies (10 and 30 Hz for **i** and **j**) during locomotor-like movement (right).

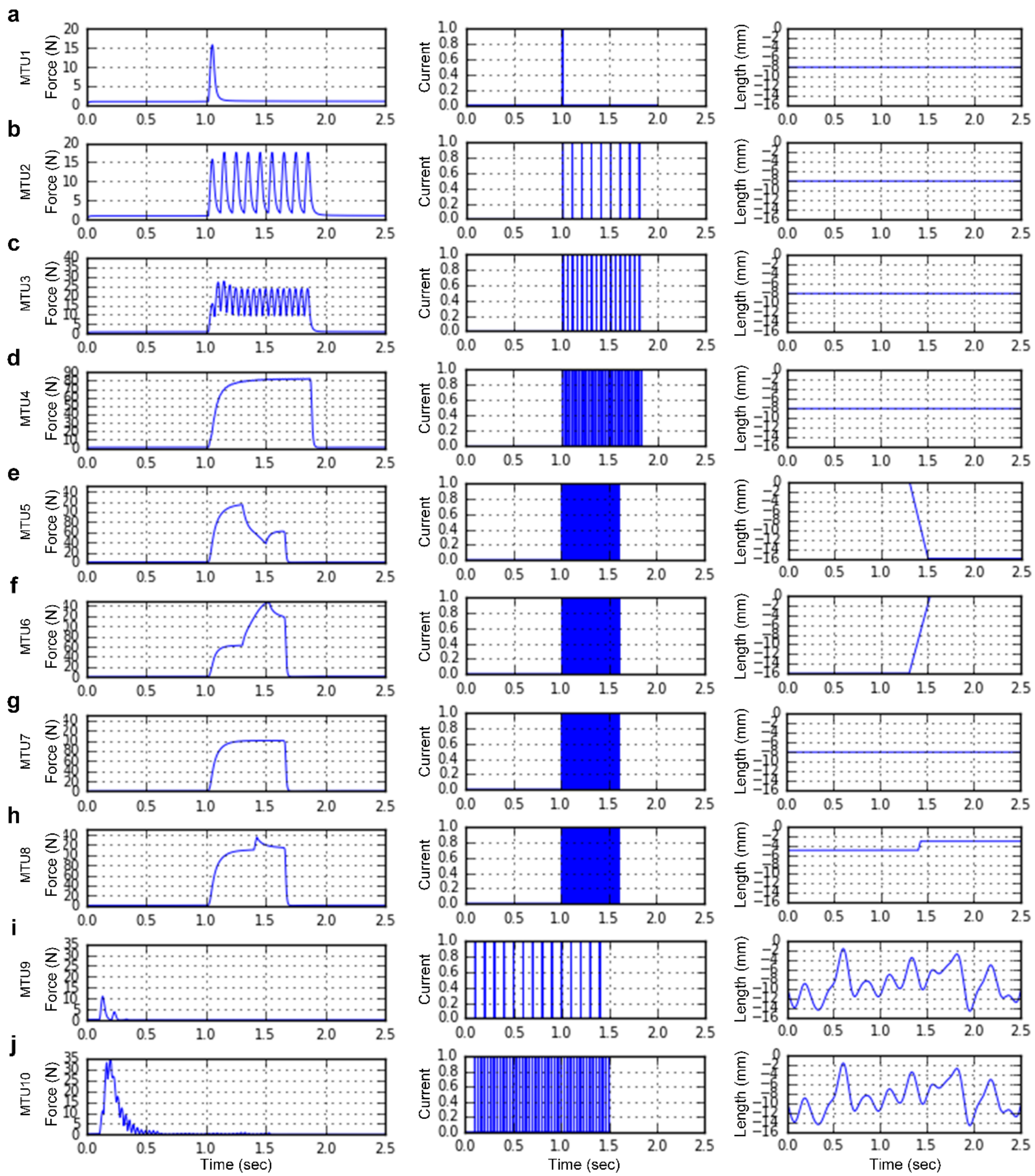

Fig D. Homogeneous fast MT population behavior. A homogenous population model consisting of 10 fast-type MTs with the same CPs was constructed and simulated as reported in a previous study (4). **a-d.** Unfused tetani of individual MTs (left) at constant stimulation frequencies (1, 10, 20 and 40 Hz for **a-d**, respectively) (middle) under isometric conditions at the intermediate length ($X_{m,0.5}$=-8 mm) (right). **e-h.** Fused tetani of individual MTs (left) under full excitation (100 Hz) (middle) during muscle shortening (**e**), lengthening (**f**), isometric (**g**), and step shortening (**h**) (right). **i-j.** Unfused tetani of

individual MTs (left) at constant stimulation frequencies (10 and 30 Hz for **i** and **j**) during locomotor-like movement (right).

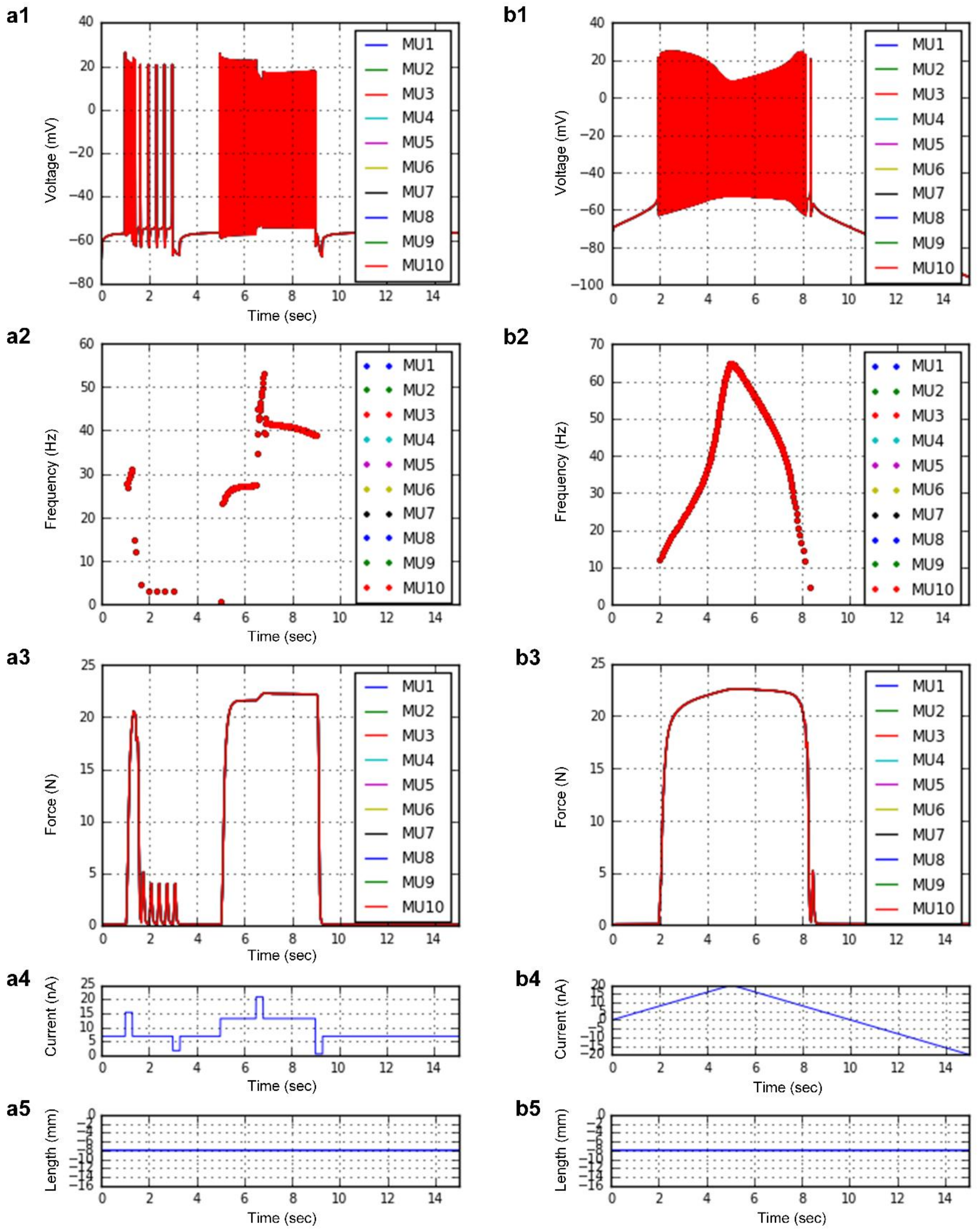

Fig E. Homogeneous MU population behavior I. A homogenous population model consisting of 10 slow-type MUs with the same CPs was constructed and simulated as reported in a previous study (5, 6). **a1-a5.** Voltage responses (**a1**) and instantaneous firing rates (**a2**) of individual MNs and force responses (**a3**) of individual MTs in

response to alternating step current (**a4**) injected intracellularly at the somata of the MNs during isometric contractions at the intermediate MT length (**a5**). **b1-b5.** Voltage responses (**b1**) and instantaneous firing rates (**b2**) of the individual MNs and force responses (**b3**) of the individual MTs in response to triangular current injection (**b4**) at the somata of the MNs during isometric contractions at the intermediate MT length (**b5**).

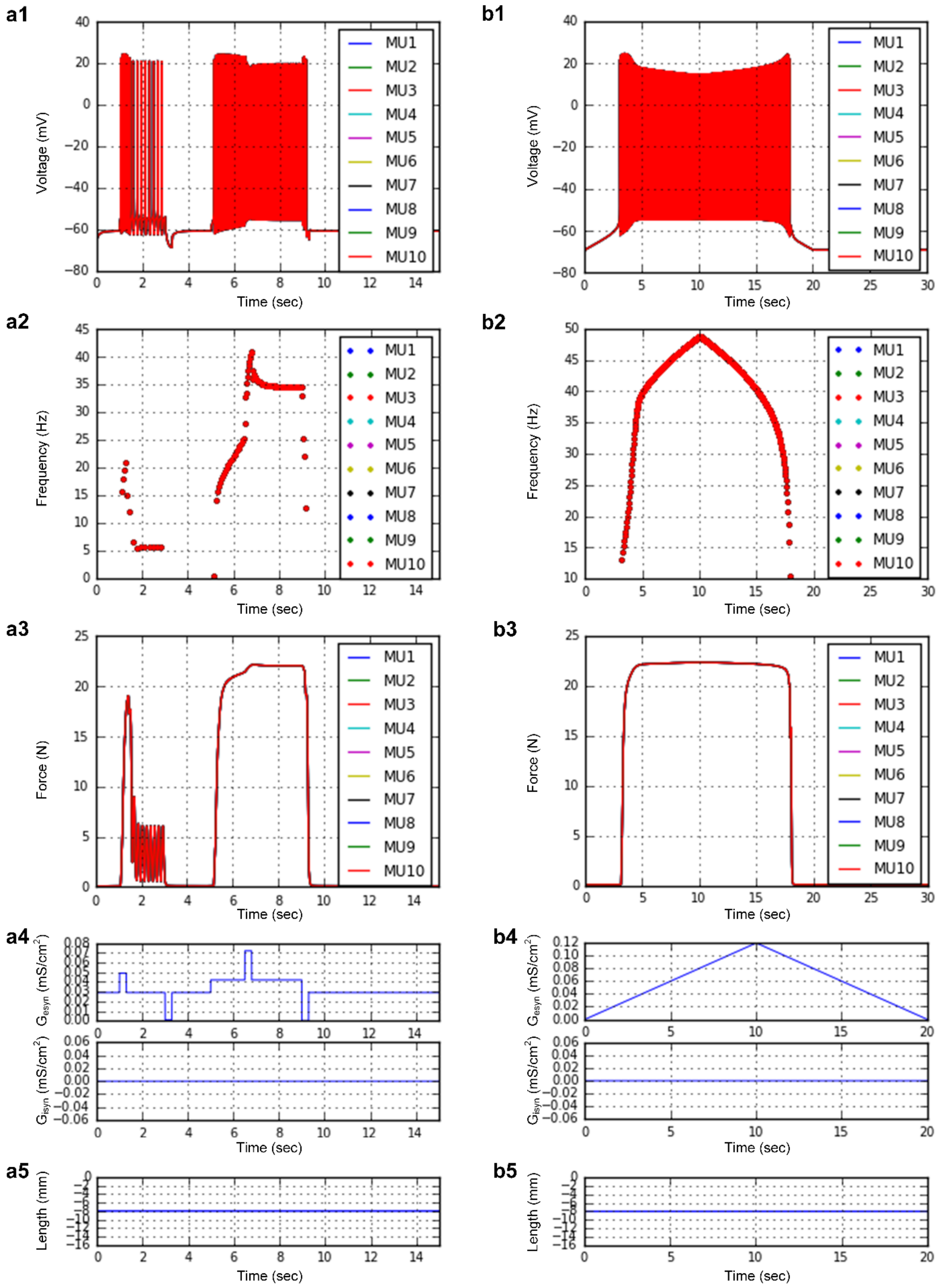
a1
Voltage (mV)
Time (sec)
MU1
MU2
MU3
MU4
MU5
MU6
MU7
MU8
MU9
MU10
b1
Voltage (mV)
Time (sec)
a2
Frequency (Hz)
Time (sec)
b2
Frequency (Hz)
Time (sec)
a3
Force (N)
Time (sec)
b3
Force (N)
Time (sec)
a4
G_{esyn} (mS/cm^2)
G_{isyn} (mS/cm^2)
Time (sec)
b4
G_{esyn} (mS/cm^2)
G_{isyn} (mS/cm^2)
Time (sec)
a5
Length (mm)
Time (sec)
b5
Length (mm)
Time (sec)

Fig F. Homogeneous MU population behavior II. The same population model used in Fig E was simulated as that reported in a previous study (5, 6). **a1-a5.** Voltage responses (**a1**) and instantaneous firing rates (**a2**) of individual MNs and force responses (**a3**) of individual MTs in response to alternating excitatory step synaptic input (**a4**) over the MN dendrites during the isometric contractions at the intermediate MT length (**a5**). **b1-b5.** Voltage responses (**b1**) and instantaneous firing rates (**b2**) of the individual MNs and force responses (**b3**) of the individual MTs in response to the excitatory triangular synaptic input (**b4**) over the MN dendrites during isometric contractions at the intermediate MT length (**b5**).

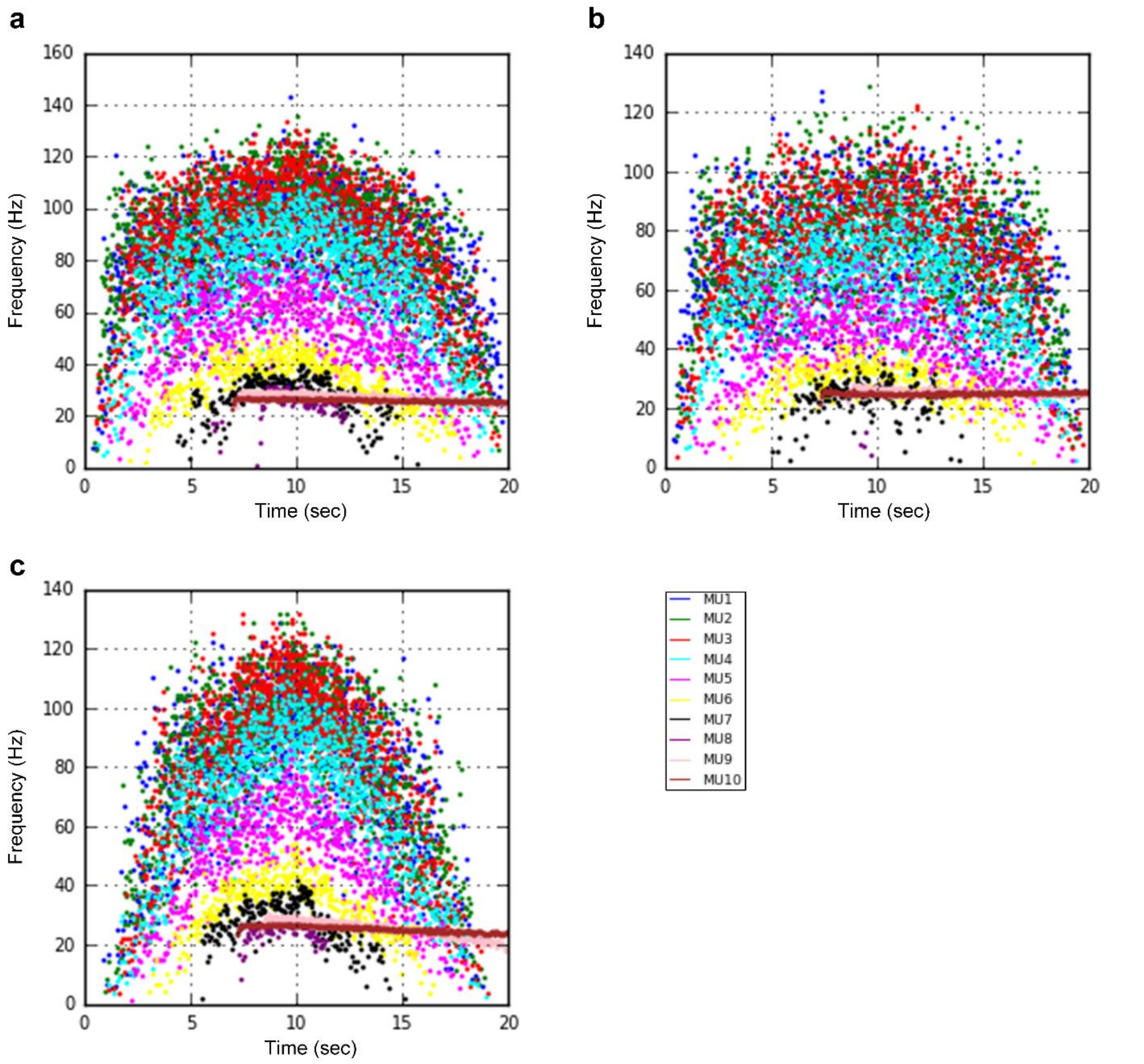

Fig G. Heterogeneous MU population behavior. Raw data for the instantaneous firing rates of individual MNs in response to the excitatory synaptic input with background inhibitory synaptic input (**a**), balanced excitatory and inhibitory synaptic input (**b**), and push-pull excitatory and inhibitory synaptic input (**c**) over the MN dendrites in Fig 10.

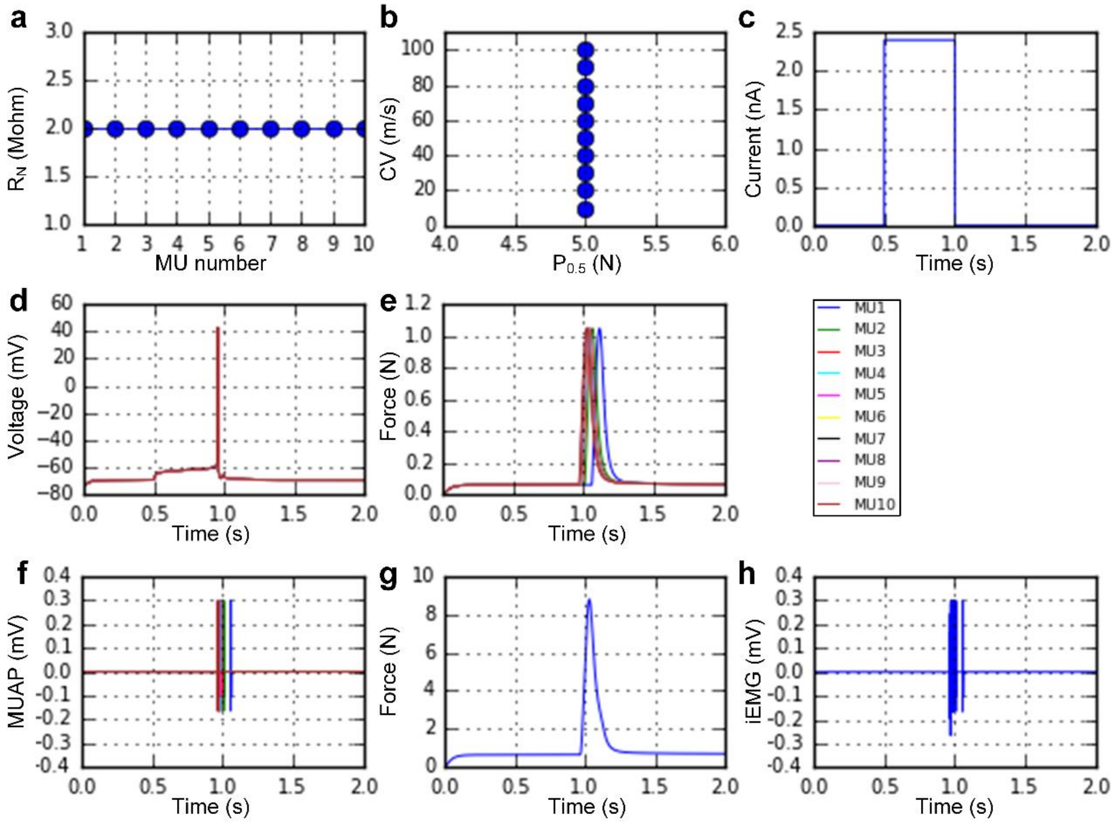

Fig H. Simulation of asynchronous activation of muscle fibers in a motor unit. a. Motoneuron input resistance (R_N) across the motor unit population. **b.** Axonal conduction velocity across the motor unit population. **c.** Intracellular input to the somata of the motoneurons. **d.** Action potentials of the motoneurons. **e.** Force responses of individual muscle fibers. **f.** MUAPs of individual muscle fibers. **g.** Force production by the entire motor unit population. **h.** Intracellular EMG (iEMG) produced by the entire motor unit population.

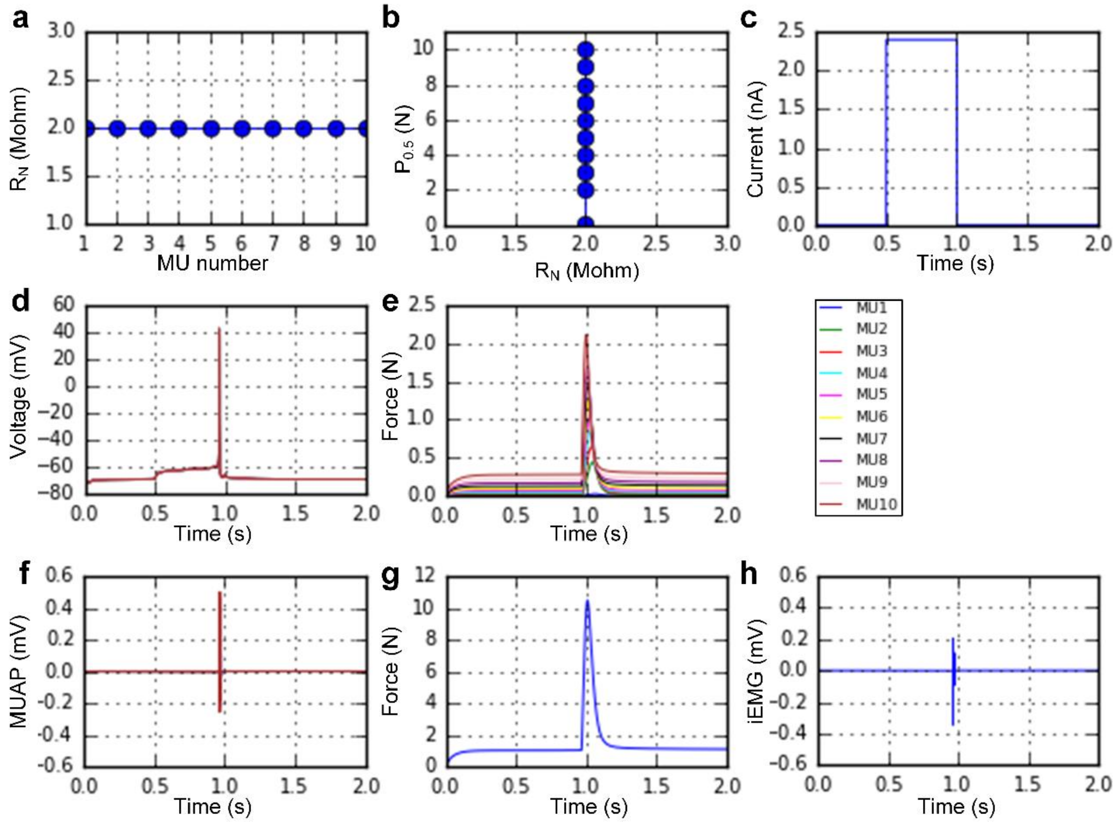

Fig I. Simulation of a motor unit with heterogeneous muscle fibers. a. Motoneuron input resistance (R_N) across the motor unit population. **b.** Muscle fiber peak force ($P_{0.5}$) across the motor unit population. **c.** Intracellular input to the somata of the motoneurons. **d.** Action potentials of the motoneurons. **e.** Force responses of individual muscle fibers. **f.** MUAPs of individual muscle fibers. **g.** Force production by the entire motor unit population. **h.** Intracellular EMG (iEMG) produced by the entire motor unit population.

Table A. Simulation engine package

Group	Element	Type	Description
Model	Cell	Class	Parent class for a cell
	Motoneuron	Class	Child class of Cell class for motoneuron
	MuscleFibers	Class	Child class of Cell class for muscle fiber
	Unit	Class	Parent class for a unit
	Motorunit	Class	Child class of Unit class for motor unit
	Pool	Class	Parent class for a population
	MotoneuronPool	Class	Child class of Pool class for motoneuron pool
	MusclefibersPool	Class	Child class of Pool class for muscle-tendon fiber population
	MotorunitPool	Class	Child class of Pool class for motor unit population
	ParamtersGenerator	Class	Independent class for the generation of the range model parameter values
Input	NeuronSignalGenerator	Class	Association class for generation of intracellular current stimulation to motoneurons
	SynConSignalGenerator	Class	Association class for the generation of synaptic conductance variation to motoneurons
	SpikeSignalGenerator	Class	Association class for the generation of current impulse stimulation to muscle unit
	MuscleLengthSignalGenerator	Class	Association class for the generation of muscle-tendon length variation
Simulation	ParallelManager	Class	Association class for parallel simulation and data management
	Job()	Function	Job content for pp server
Analysis	detect_Spike()	Function	Detection of the spikes generated from the motoneuron model

	cal_FiringRate()	Function	Calculation of the firing rate of the motoneuron model

Table B. Notations of variables and parameters in the equations, codes, and result files for MN model.

Property	Equation	Code	File	Unit	Remark
Membrane potential	V_S	vs	V_soma	mV	Variable (state)
	V_D	vd	V_dend	mV	Variable (state)
	t	dt	Time	ms	Variable
	I_S	I_s	***Is	μA/cm^2	Variable
Cable property	R_N	RN		MΩ	Property (0.4~4.0)
	τ_m	tm		ms	Property
	${VA_{SD}}^{DC}$	VAsdDC			Property
	${VA_{DS}}^{DC}$	VAdsDC			Property
	${VA_{SD}}^{AC}$	VAsdAC			Property
	D_{path}	Dpath		mm	Property (0.1~1.1)
	p	parea			Constant
	ω	w		rad/sec	Constant
	$G_{m,S}$	gms		mS/cm^2	*Dependent parameter (0.005~0.762)
	$G_{m,D}$	gmd		mS/cm^2	*Dependent parameter (0.039~1.84)
	G_C	gc		mS/cm^2	*Dependent parameter (0.01~1.242)
	$C_{m,S}$	cms		μF/cm^2	*Dependent parameter (0.125~4.422)
	$C_{m,D}$	cmd		μF/cm^2	*Dependent parameter (0.234~1.136)
	$E_{Leak,S}$	svl		mV	Parameter
	$E_{Leak,D}$	dvl		mV	Parameter
Active property for the soma	$[Ca^{2+}]_{i,S}$	sca	[Ca]_soma	μM	Variable (state)
	f_S	sf			*Parameter (0.005~0.2)
	$K_{Ca,S}$	skca		ms^{-1}	Parameter
	α_S	salpha		mol/μC/cm^2	Parameter
	R	R		VCmol^{-1}k^{-1}	Constant
	T	Temp		K	Constant

	Z_{Ca}	Zca			Constant
	F	Fe		$Cmol^{-1}$	Constant
	$[Ca^{2+}]_{o,S}$	sCAo		mM	Parameter
	$E_{Ca,S}$	svca	E_Ca_soma	mV	**Variable
	$I_{Naf,S}$	sina	I_Naf_soma	$\mu A/cm^2$	Variable
	$G_{Naf,S}$	sgna		mS/cm^2	*Parameter (30.7~144.5)
	$E_{Na,S}$	svna		mV	Parameter
	m_{naf}	snam	m_Naf_soma		Variable (state)
	h_{naf}	snah	h_Naf_soma		Variable (state)
	α_m	alpha_snafm			Variable
	β_m	beta_snafm			Variable
	h_∞	snahinf			Variable
	τ_h	snahtau			Variable
	$\alpha_{nafm1,S}$	sanamc		$(mV\cdot ms)^{-1}$	Parameter
	$\alpha_{nafm2,S}$	sanamv		mV	Parameter
	$\alpha_{nafm3,S}$	sanama		mV	Parameter
	$\alpha_{nafm4,S}$	sanamb			Parameter
	$\beta_{nafm1,S}$	sbnamc		$(mV\cdot ms)^{-1}$	Parameter
	$\beta_{nafm2,S}$	sbnamv		mV	Parameter
	$\beta_{nafm3,S}$	sbnama		mV	Parameter
	$\beta_{nafm4,S}$	sbnamb			Parameter
	$\gamma_{nafh1,S}$	snahth		mV	Parameter
	$\gamma_{nafh2,S}$	snahslp		mV	Parameter
	$\gamma_{nafh3,S}$	snahv		mV	Parameter
	$\gamma_{nafh4,S}$	snaha		mV	Parameter
	$\gamma_{nafh5,S}$	snahb		mV	Parameter
	$\gamma_{nafh6,S}$	snahc		mV	Parameter
	$I_{Kdr,S}$	sikdr	I_Kdr_soma	$\mu A/cm^2$	Variable
	$G_{Kdr,S}$	sgkdr		mS/cm^2	Parameter
	$E_{K,S}$	svk		mV	Parameter
	n_{kdr}	skdr	n_Kdr_soma		Variable (state)
	n_∞	skdrinf			Variable
	τ_n	skdrtau			Variable
	$\gamma_{kdrm1,S}$	skdrth		mV	Parameter
	$\gamma_{kdrm2,S}$	skdrslp		mV	Parameter
	$\gamma_{kdrm3,S}$	skdrv		mV	Parameter
	$\gamma_{kdrm4,S}$	skdra		mV	Parameter
	$\gamma_{kdrm5,S}$	skdrb		mV	Parameter
	$\gamma_{kdrm6,S}$	skdrc		ms	Parameter

	$I_{Can,S}$	sica	I_Can_soma	μA/cm^2	Variable
	$G_{Can,S}$	sgca		mS/cm^2	Parameter
	m_{can}	scam	m_Can_soma		Variable (state)
	h_{can}	scah	h_Can_soma		Variable (state)
	m_{∞}	scaminf			Variable
	h_{∞}	scahinf			Variable
	$\gamma_{canm1,S}$	scamth		mV	Parameter
	$\gamma_{canm2,S}$	scamslp		mV	Parameter
	$\gamma_{canm3,S}$	scamtau		ms	Parameter
	$\gamma_{canh1,S}$	scahth		mV	Parameter
	$\gamma_{canh2,S}$	scahslp		mV	Parameter
	$\gamma_{canh3,S}$	scahtau		ms	Parameter
	$I_{K(Ca),S}$	sikca	I_Kca_soma	μA/cm^2	Variable
	$G_{K(Ca),S}$	sgkca		mS/cm^2	Parameter
	$E_{K,S}$	svk		mV	Parameter
	$K_{d,S}$	skd		mM	Parameter
	$I_{Nap,S}$	sinap	I_Nap_soma	μA/cm^2	Variable
	$G_{Nap,S}$	sgnap		mS/cm^2	Parameter
	m_{nap}	snapm	m_Nap_soma		Variable (state)
	α_m	alpha_snapm			Variable
	β_m	beta_snapm			Variable
	$\alpha_{napm1,S}$	sanapmc		(mV·ms)$^{-1}$	Parameter
	$\alpha_{napm2,S}$	sanapmv		mV	Parameter
	$\alpha_{napm3,S}$	sanapma		mV	Parameter
	$\alpha_{napm4,S}$	sanapmb			Parameter
	$\beta_{napm1,S}$	sbnapmc		(mV·ms)$^{-1}$	Parameter
	$\beta_{napm2,S}$	sbnapmv		mV	Parameter
	$\beta_{napm3,S}$	sbnapma		mV	Parameter
	$\beta_{napm4,S}$	sbnapmb			Parameter
	$I_{H,S}$	sih	I_H_soma	μA/cm^2	Variable
	$G_{H,S}$	sgh		mS/cm^2	Parameter
	$E_{H,S}$	svh		mV	Parameter
	m_h	shm	m_H_soma		Variable (state)
	m_{∞}	shminf			Variable
	$\gamma_{hm1,S}$	shth		mV	Parameter
	$\gamma_{hm2,S}$	shslp		mV	Parameter

	$\gamma_{hm3,S}$	shtau		ms	Parameter
	$I_{syn,S}$	sisyn			Variable
	$I_{esyn,S}$	siesyn	I_esyn_soma	μA/cm^2	Variable
	$I_{isyn,S}$	siisyn	I_isyn_soma	μA/cm^2	Variable
	$G_{esyn,S}$	sgesyn	G_esyn_soma	mS/cm^2	Variable
	$G_{isyn,S}$	sgisyn	G_isyn_soma	mS/cm^2	Variable
	$G_{esyn0,S}$	sgesyn0		mS/cm^2	Variable
	$G_{isyn0,S}$	sgisyn0		mS/cm^2	Variable
	σ_{esyn}	esynsigma		mS/cm^2	Variable
	σ_{isyn}	isynsigma		mS/cm^2	Variable
	$\tau_{esyn,S}$	sesyntau		ms	Parameter
	$\tau_{isyn,S}$	sisyntau		ms	Parameter
	$E_{esyn,S}$	svesyn		mV	Parameter
	$E_{isyn,S}$	svisyn		mV	Parameter
Active property for the dendrite	$[Ca^{2+}]_{i,D}$	dca	[Ca]_dend	μM	Variable (state)
	f_D	df			Parameter
	$K_{Ca,D}$	dkca		ms^{-1}	Parameter
	α_D	dalpha		mol/μC/cm^2	Parameter
	R	R		VCmol^{-1}k^{-1}	Constant
	T	Temp		K	Constant
	Z_{Ca}	Zca			Constant
	F	Fe		Cmol^{-1}	Constant
	$[Ca^{2+}]_{o,D}$	dCAo		mM	Parameter
	$E_{Ca,D}$	dvca	E_Ca_dend	mV	**Variable
	$I_{Naf,D}$	dina	I_Naf_dend	μA/cm^2	Variable
	$G_{Naf,D}$	dgna		mS/cm^2	Parameter
	$E_{Na,D}$	dvna		mV	Parameter
	m_{naf}	dnam	m_Naf_dend		Variable (state)
	h_{naf}	dnah	h_Naf_dend		Variable (state)
	α_m	alpha_dnafm			Variable
	β_m	beta_dnafm			Variable
	h_∞	dnahinf			Variable
	τ_h	dnahtau			Variable
	$\alpha_{nafm1,D}$	danamc		(mV·ms)$^{-1}$	Parameter
	$\alpha_{nafm2,D}$	danamv		mV	Parameter
	$\alpha_{nafm3,D}$	danama		mV	Parameter
	$\alpha_{nafm4,D}$	danamb			Parameter
	$\beta_{nafm1,D}$	dbnamc		(mV·ms)$^{-1}$	Parameter

	$\beta_{nafm2,D}$	dbnamv		mV	Parameter
	$\beta_{nafm3,D}$	dbnama		mV	Parameter
	$\beta_{nafm4,D}$	dbnamb			Parameter
	$\gamma_{nafh1,D}$	dnahth		mV	Parameter
	$\gamma_{nafh2,D}$	dnahslp		mV	Parameter
	$\gamma_{nafh3,D}$	dnahv		mV	Parameter
	$\gamma_{nafh4,D}$	dnaha		mV	Parameter
	$\gamma_{nafh5,D}$	dnahb		mV	Parameter
	$\gamma_{nafh6,D}$	dnahc		mV	Parameter
	$I_{Kdr,D}$	dikdr	I_Kdr_dend	$\mu A/cm^2$	Variable
	$G_{Kdr,D}$	dgkdr		mS/cm^2	Parameter
	$E_{K,D}$	dvk		mV	Parameter
	n_{kdr}	dkdr	n_Kdr_dend		Variable (state)
	n_{∞}	dkdrinf			Variable
	τ_n	dkdrtau			Variable
	$\gamma_{kdrm1,D}$	dkdrth		mV	Parameter
	$\gamma_{kdrm2,D}$	dkdrslp		mV	Parameter
	$\gamma_{kdrm3,D}$	dkdrv		mV	Parameter
	$\gamma_{kdrm4,D}$	dkdra		mV	Parameter
	$\gamma_{kdrm5,D}$	dkdrb		mV	Parameter
	$\gamma_{kdrm6,D}$	dkdrc		ms	Parameter
	$I_{Can,D}$	dica	I_Can_dend	$\mu A/cm^2$	Variable
	$G_{Can,D}$	dgca		mS/cm^2	Parameter
	m_{can}	dcam	m_Can_dend		Variable (state)
	h_{can}	dcah	h_Can_dend		Variable (state)
	m_{∞}	dcaminf			Variable
	h_{∞}	dcahinf			Variable
	$\gamma_{canm1,D}$	dcamth		mV	Parameter
	$\gamma_{canm2,D}$	dcamslp		mV	Parameter
	$\gamma_{canm3,D}$	dcamtau		ms	Parameter
	$\gamma_{canh1,D}$	dcahth		mV	Parameter
	$\gamma_{canh2,D}$	dcahslp		mV	Parameter
	$\gamma_{canh3,D}$	dcahtau		ms	Parameter
	$I_{K(Ca),D}$	dikca	I_Kca_dend	$\mu A/cm^2$	Variable
	$G_{K(Ca),D}$	dgkca		mS/cm^2	*Parameter (0.001~0.15)
	$E_{K,D}$	dvk		mV	Parameter

	m_{kca}	dkcam	m_Kca_dend		Variable (state)
	$\gamma_{kcam1,D}$	dkcamth		mM	Parameter
	$\gamma_{kcam2,D}$	dkcamslp			Parameter
	$\gamma_{kcam3,D}$	dkcamtau		ms	Parameter
	$I_{Nap,D}$	dinap	I_Nap_dend	μA/cm^2	Variable
	$G_{Nap,D}$	dgnap		mS/cm^2	Parameter
	$E_{Na,D}$	dvna		mV	Parameter
	m_{nap}	dnapm	m_Nap_dend		Variable (state)
	α_m	alpha_dnapm			Variable
	β_m	beta_dnapm			Variable
	$\alpha_{napm1,D}$	danapmc		(mV·ms)$^{-1}$	Parameter
	$\alpha_{napm2,D}$	danapmv		mV	Parameter
	$\alpha_{napm3,D}$	danapma		mV	Parameter
	$\alpha_{napm4,D}$	danapmb			Parameter
	$\beta_{napm1,D}$	dbnapmc		(mV·ms)$^{-1}$	Parameter
	$\beta_{napm2,D}$	dbnapmv		mV	Parameter
	$\beta_{napm3,D}$	dbnapma		mV	Parameter
	$\beta_{napm4,D}$	dbnapmb			Parameter
	$I_{Cal,D}$	dical	I_Cal_dend	μA/cm^2	Variable
	$G_{Cal,D}$	dgcal		mS/cm^2	*Parameter (0.104 ~ 3.15)
	S_{nm}	SNM			*Parameter (0.1 ~ 10)
	l_{cal}	dcal	m_Cal_dend		Variable (state)
	$\gamma_{calm1,D}$	dcalth		mV	Parameter
	$\gamma_{calm2,D}$	dcalslp		mV	Parameter
	$\gamma_{calm3,D}$	dcaltau		ms	Parameter
	$I_{H,D}$	dih	I_H_dend	μA/cm^2	Variable
	$G_{H,D}$	dgh		mS/cm^2	Parameter
	$E_{H,D}$	dvh		mV	Parameter
	m_h	dhm	m_H_dend		Variable (state)
	m_∞	dhminf			Variable
	$\gamma_{hm1,D}$	dhth		mV	Parameter
	$\gamma_{hm2,D}$	dhslp		mV	Parameter
	$\gamma_{hm3,D}$	dhtau		ms	Parameter
	$I_{syn,D}$	disyn			Variable

	$I_{esyn,D}$	diesyn	I_esyn_dend	μA/cm^2	Variable
	$I_{isyn,D}$	diisyn	I_isyn_dend	μA/cm^2	Variable
	$G_{esyn,D}$	dgesyn	G_esyn_dend	mS/cm^2	Variable
	$G_{isyn,D}$	dgisyn	G_isyn_dend	mS/cm^2	Variable
	$G_{esyn0,D}$	dgesyn0		mS/cm^2	Variable
	$G_{isyn0,D}$	dgisyn0		mS/cm^2	Variable
	σ_{esyn}	esynsigma		mS/cm^2	Variable
	σ_{isyn}	isynsigma		mS/cm^2	Variable
	$\tau_{esyn,D}$	desyntau		ms	Parameter
	$\tau_{isyn,D}$	disyntau		ms	Parameter
	$E_{esyn,D}$	dvesyn		mV	Parameter
	$E_{isyn,D}$	dvisyn		mV	Parameter
Signal transmission for the axon	CV	cv		m·s^{-1}	*Parameter (57 ~ 117)

* Parameter indicates the range model parameter whose value is varied across the heterogeneous population model. The values of five dependent parameters are determined directly from the five electrotonic properties ($R_{N,S}$, τ_m, VA_{SD}^{DC}, VA_{DS}^{DC}, VA_{SD}^{AC}).
** $E_{Ca,S}$ and $E_{Ca,D}$ can be dynamically varied or constant. This can be selected in the parameter file by assigning sEca and dEca to be zero or a desired value.
*** I_S in Equation was multiplied by a conversion factor of 3.1576*10^{-3} cm^2 when saved in a unit of nA in the result file. The conversion factor corresponds to the surface area accumulated from the cell body to the dendritic sites separated by 0.6 mm from the soma in a fully reconstructed motoneuron (cell number 43/5 (FR-type) in (7)). The morphology (v_e_moto6) of the motoneuron was downloaded from http://NeuroMorpho.Org.
The numbers in the parenthesis indicate the minimum and maximum of the range of parameter values observed in previous cat studies (8).

Table C. Notations of variables and parameters in the equations, codes, and result files for MT model.

Module	Equation	Code	File	Unit	Remark
MODULE 1	t	dt	Time	ms	variable
	X_m	Xm	Xm	mm	Variable
	V_m	Vm	Vm	$mm{\cdot}ms^{-1}$	variable
	A_m	Am	Am	$mm{\cdot}ms^{-2}$	variable
	$[Ca_{SR}]$	CaSR	CaSR	M	Variable (state)
	$[Ca_{SP}]$	CaSP	CaSP	M	Variable (state)
	$[Ca_{SR}CS]$	CaSRCS	CaSRCS	M	Variable (state)
	$[Ca_{SP}B]$	CaSPB	CaSPB	M	Variable (state)
	$[Ca_{SP}T]$	CaSPT	CaSPT	M	Variable (state)
	R	R	R	$M{\cdot}ms^{-1}$	Variable
	U	U		$M{\cdot}ms^{-1}$	Variable
	$K5$	K5		$M^{-1}{\cdot}ms^{-1}$	Variable
	$K6$	K6		ms^{-1}	Variable
	$\varphi(X_m)$	uXm			Variable
	$K1$	K1		$M^{-1}{\cdot}ms^{-1}$	Parameter
	$K2$	K2		ms^{-1}	Parameter
	$K3$	K3		$M^{-1}{\cdot}ms^{-1}$	Parameter
	$K4$	K4		ms^{-1}	Parameter
	$K5_i$	K5i		$M^{-1}{\cdot}ms^{-1}$	Parameter
	$K6_i$	K6i		ms^{-1}	Parameter
	K	K		M^{-1}	Parameter
	R_{max}	Rmax		ms^{-1}	Parameter
	U_{max}	Umax		$M{\cdot}ms^{-1}$	Parameter
	τ_1	tau1		ms	*Parameter (3~1)
	τ_2	tau2		ms	*Parameter (25~13)
	φ_1	phi1		mm^{-1}	*Parameter (0.03~0.002)
	φ_2	phi2			*Dependent parameter
	φ_3	phi3		mm^{-1}	*Parameter (0.01~10^{-4})

	φ_4	phi4			*Dependent parameter
MODULE 2	A	As	A		Variable (state)
	$\tilde{A}$	A	A_tilde		Variable
	$\tilde{A}_\infty$	Ainf			Variable
	C1	C1	C1		Variable (state)
	C2	C2	C2		Variable (state)
	$\tau_{\tilde{A}}$	TA			Variable
	CS_0	CS0			Parameter
	B_0	B0			Parameter
	T_0	T0			Parameter
	c1i	C1i			*Parameter (0.12~0.16)
	c1n1	C1n1			*Parameter (0~0.01)
	c1n2	C1n2			Parameter
	c1n3	C1n3			Parameter
	c1n4	C1n4		ms	*Parameter (0.0001~85)
	c2i	C2i			*Parameter (0.09~0.15)
	c2n1	C2n1			*Parameter (0~-0.04)
	c2n2	C2n2			Parameter
	c2n3	C2n3			Parameter
	c2n4	C2n4		ms	*Parameter (0.0001~70)
	C3	C3		ms	*Parameter (62~54)
	C4	C4			*Parameter (-13~-19)
	C5	C5			*Parameter (5.1~3.9)
	α_i	alpha_i			*Parameter (2~1.6)
	α_1	a1			Parameter
	α_2	a2			Parameter
	α_3	a3			Parameter
	β	beta			*Parameter (0.5~0.09)

	γ	gamma			*Parameter (0.001~0.0002)
MODULE 3	F	F	F		Variable
	X_{CE}	XCE	XCE		Variable
	$g(X_m)$	gXm			Variable
	K_{SE}	KSE		mm^{-1}	*Parameter (0.4~0.16)
	$P_{0.5}$	P0		N	*Parameter (0.1~10)
	g_1	g1		mm	*Parameter (-8~-0.8)
	g_2	g2		mm	*Parameter (22~17)
	g_3	g3			*Dependent parameter
	a_0	a0			*Parameter (0.1~0.004)
	b_0	b0		$mm \cdot s^{-1}$	*Parameter (24~100)
	c_0	c0			*Parameter (-0.32~-0.58)
	d_0	d0		$mm \cdot s^{-1}$	*Parameter (30~43)
Intramuscular EMG	$MUAP$	MUAP	MUAP	μV	Variable
	A_{MUAP}	AM		μV	*Parameter (0.1~0.5)
	L_{MUAP}	LM		ms	*Parameter (1.0~0.5)
Axonal nerve	CV	cv		$m \cdot s^{-1}$	*Parameter (57~117)

* Parameter indicates the range model parameter whose value is varied across the heterogeneous population model. The dependent parameters of φ_2 and φ_4 in module 1 and g_3 in module 3 are determined directly from the values of φ_1 and φ_3 in module 1 and g_1 and g_2 in module 3 (see Appendix B in S3 Text for the details). The numbers in the parenthesis indicate the range of parameter values obtained from the soleus and medial gastrocnemius muscles in previous cat studies (3, 4), except for the A_{MUAP}, L_{MUAP}, and CV whose values were adopted from earlier studies (9, 10).

References:

1. Kim H, Jones KE, Heckman CJ. Asymmetry in signal propagation between the soma and dendrites plays a key role in determining dendritic excitability in motoneurons. PloS one. 2014;9(8):e95454.
2. Kim H. Impact of the localization of dendritic calcium persistent inward current on the input-output properties of spinal motoneuron pool: a computational study. Journal of applied physiology. 2017;123(5):1166-87.
3. Kim H, Sandercock TG, Heckman CJ. An action potential-driven model of soleus muscle activation dynamics for locomotor-like movements. J Neural Eng. 2015;12(4):046025.
4. Kim H, Heckman CJ. A dynamic calcium-force relationship model for sag behavior in fast skeletal muscle. PLoS Comput Biol. 2023;19(6):e1011178.
5. Kim H, Kim M. PyMUS: Python-Based Simulation Software for Virtual Experiments on Motor Unit System. Front Neuroinform. 2018;12:15.
6. Kim H. Muscle length-dependent contribution of motoneuron Cav1.3 channels to force production in model slow motor unit. Journal of applied physiology. 2017;123(1):88-105.
7. Cullheim S, Fleshman JW, Glenn LL, Burke RE. Membrane area and dendritic structure in type-identified triceps surae alpha motoneurons. J Comp Neurol. 1987;255(1):68-81.
8. Zengel JE, Reid SA, Sypert GW, Munson JB. Membrane electrical properties and prediction of motor-unit type of medial gastrocnemius motoneurons in the cat. J Neurophysiol. 1985;53(5):1323-44.
9. Cisi RR, Kohn AF. Simulation system of spinal cord motor nuclei and associated nerves and muscles, in a Web-based architecture. Journal of computational neuroscience. 2008;25(3):520-42.
10. Hoffer JA, Loeb GE, Marks WB, O'Donovan MJ, Pratt CA, Sugano N. Cat hindlimb motoneurons during locomotion. I. Destination, axonal conduction velocity, and recruitment threshold. J Neurophysiol. 1987;57(2):510-29.
