# Supplementary material for: Automating population construction and parallel simulation of biophysical models for neuromuscular cells: An inverse approach
Source: PLoS Comput Biol. 2026 Apr 24;22(4):e1014184. doi: 10.1371/journal.pcbi.1014184 (PMC13132451; doi:10.1371/journal.pcbi.1014184)
Supplement: S2 Text — (PDF) [file pcbi.1014184.s002.pdf]

User manual for the pNMS 3.0 API

1 How to use pNMS API

```
# import API library from the pNMS folder
import pNMS.application_programming as cf

# use API functions
cf.function_name(…)
```

2 pNMS API functions

Creation of a homogeneous population model
- createPool

Transition to a heterogeneous population model
- setParameters
- plot_params

Setup of simulation conditions
- setInitialValues
- setSimulTimes

Setup of input conditions
- genNeuronInputSignals
- genSynConSignals
- genSpikeSignals
- genMuscleLengthSignals
- importNeuronInputSignals
- importSynConSignals
- importSpikeSignals
- importMuscleLengthSignals
- setNeuronInputSignals
- setSynConSignals
- setSpikeSignals
- setMuscleLengthSignals
- plotNeuronInputSignal
- plotSynConSignal
- plotSpikeSignal
- plotMuscleLengthSignal

Setup of parallel computing environment
- setComputeNode

Execution of parallel simulation
- runSimulation

Display and saving of simulation results
- plotSimulResult
- saveSimulationResults
- plotImportData

3 API function description

createPool*(pool_type='motorunit', num=10)*

Creates a homogeneous population instance comprising a specified number of cell or unit models having the same parameter values through importing the parameter file.

Parameters:

pool_type: *{'motoneuron', 'musclefibers', 'motorunit'}, default 'motorunit'*
The model type to be created.

pool_count : *int, default 10*
The number of models (1 ~ #).

setParameters*(param_file, MF_file=None, RN_file=None, Dpath_file=None, gms_file=None, gmd_file=None, gc_file=None, cms_file=None, cmd_file=None, sf_file=None, sgna_file=None, dgcal_file=None, dgkca_file=None, SNM_file=None, p0_file=None, tau1_file=None, tau2_file=None, KSE_file=None, AM_file=None, LM_file=None, cv_file=None, phi1_file=None, phi3_file=None, C1i_file=None, C1n1_file=None, C1n4_file=None, C2i_file=None, C2n1_file=None, C2n4_file=None, C3_file=None, C4_file=None, C5_file=None, alpha_i_file=None, beta_file=None, gamma_file=None, g1_file=None, g2_file=None, a0_file=None, b0_file=None, c0_file=None, d0_file=None, RN_func=None, Dpath_func=None, gms_func=None, gmd_func=None, gc_func=None, cms_func=None, cmd_func=None, sf_func=None, sgna_func=None, dgcal_func=None, dgkca_func=None, SNM_func=None, p0_func=None, tau1_func=None, tau2_func=None, KSE_func=None, AM_func=None, LM_func=None, cv_func=None, phi1_func=None, phi3_func=None, C1i_func=None, C1n1_func=None, C1n4_func=None, C2i_func=None, C2n1_func=None, C2n4_func=None, C3_func=None, C4_func=None, C5_func=None, alpha_i_func=None, beta_func=None, gamma_func=None, g1_func=None, g2_func=None, a0_func=None, b0_func=None, c0_func=None, d0_func=None, RN_params=None, Dpath_params=None, gms_params=None, gmd_params=None, gc_params=None, cms_params=None, cmd_params=None, sf_params=None, sgna_params=None, dgcal_params=None, dgkca_params=None, SNM_params=None, p0_params=None, tau1_params=None, tau2_params=None, KSE_params=None, AM_params=None, LM_params=None, cv_params=None, phi1_params=None, phi3_params=None, C1i_params=None, C1n1_params=None, C1n4_params=None, C2i_params=None, C2n1_params=None, C2n4_params=None, C3_params=None, C4_params=None, C5_params=None, alpha_i_params=None, beta_params=None, gamma_params=None, g1_params=None, g2_params=None, a0_params=None, b0_params=None, c0_params=None, d0_params=None, RN_range=None, Dpath_range=None, gms_range=None, gmd_range=None, gc_range=None, cms_range=None, cmd_range=None, sf_range=None, sgna_range=None, dgcal_range=None, dgkca_range=None, SNM_range=None, p0_range=None, tau1_range=None, tau2_range=None, KSE_range=None, AM_range=None, LM_range=None, cv_range=None, phi1_range=None, phi3_range=None, C1i_range=None, C1n1_range=None, C1n4_range=None, C2i_range=None, C2n1_range=None, C2n4_range=None, C3_range=None, C4_range=None, C5_range=None, alpha_i_range=None, beta_range=None, gamma_range=None, g1_range=None, g2_range=None, a0_range=None, b0_range=None, c0_range=None, d0_range=None, init=False)*

Converts the homogeneous population instance into the heterogeneous population instance by setting the range parameter values to systematically vary across the population instance through importing the user-defined data or using the built-in functions.

Parameters:

param_file: *str*

Path to the parameter file for the model type of motoneuron, musclefibers, and motor unit (e.g., '../parameters/MN_Parameters/MN_Parameters_2.1.3.csv').

MF_file: *str, optional for the model type of motorunit*

Path to the parameter file for the model type of musclefibers (e.g., '../parameters/MF_Parameters/MF_Parameters_2.1.3.csv').

Range parameter_file: *str*, *optional*

Path to the user-defined data for the distribution of range parameter value (e.g., '../parameters/MN_Parameters/MN_Parameters_2.1.3.csv').
RN, Dpath, gms, gmd, gc, cms, cmd, sgna, sf, dgcal, dgkca, SNM for the model type of motoneuron and motorunit.
p0, tau1, tau2, KSE, AM, LM, cv, phi1, phi3, C1i, C1n1, C1n4, C2i, C2n1, C2n4, C3, C4, C5, alpha_i, beta, gamma, g1, g2, a0, b0, c0, d0 for the model type of musclefibers.

Range parameter_func: *{'linear', 'inc_convex', 'inc_concave', 'dec_convex', 'dec_concave'}, optional*

Use of built-in function for the distribution of range parameter value.
RN, Dpath, gms, gmd, gc, cms, cmd, sgna, sf, dgcal, dgkca, SNM for the model type of motoneuron and motorunit.
p0, tau1, tau2, KSE, AM, LM, cv, phi1, phi3, C1i, C1n1, C1n4, C2i, C2n1, C2n4, C3, C4, C5, alpha_i, beta, gamma, g1, g2, a0, b0, c0, d0 for the model type of musclefibers and motorunit.

Range parameter_params: *list, optional*

Array of parameter values for the built-in function.
[slope, y-intercept] for the 'linear' straight line function.
[start of x-domain, end of x-domain] for the 'inc_convex' 'inc_convex', 'inc_concave', 'dec_convex', and 'dec_concave' exponential function.

Range parameter_range: *list, optional*

Array of parameter values for the nonlinear built-in functions ('inc_convex' 'inc_convex', 'inc_concave', 'dec_convex', and 'dec_concave').
[minimum, maximum] for the range of range parameter values.

Init: *bool, default False*

Indicator of heterogeneity in range parameter values across the population instance.

plot_params*(param, unitType=None)*

Plots the distribution of the range parameter values for the heterogeneous population instance.

Parameters:

param: *{'RN', 'Dpath', 'gms', 'gmd', 'gc', 'cms', 'cmd', 'sf', 'sgna', 'dgcal', 'SNM', 'dgkca'}* for the model type of motoneuron and motorunit and *{'p0', 'tau1', 'tau2', 'KSE', 'AM', 'LM', 'cv', 'phi1', 'phi3', 'C1i', 'C1n1', 'C1n4', 'C2i', 'C2n1', 'C2n4', 'C3', 'C4', 'C5', 'alpha_i', 'beta', 'gamma', 'g1', 'g2', 'a0', 'b0', 'c0', 'd0'}* for the model type of musclefibers and motorunit.

type: *{'motorunit'}, optional for the model type of motorunit*

Plot of RN-MU# and P0-RN relationship.

setInitialValues*(ivalue_1, ivalue_2=None)*

Sets the initial values of the state variables.

Parameters:

ivalue_1: *list*

[vs, sca, snam, snah, snapm, skdr, scam, scah, shm, vd, dca, dcal, dnam, dnah, dnapm, dkdr, dkcam, dcam, dcah, dhm] for the model type of motoneuron and motorunit.

[CaSR, CaSRCS, CaSP, CaSPB, CaSPT, C1, C2, A, XCE] for the model type of musclefibers.

ivalue_2: *list, optional for the model type of motorunit*

[CaSR, CaSRCS, CaSP, CaSPB, CaSPT, C1, C2, A, XCE] for the muscle-tendon unit model.

setSimulTimes*(t_start=0, t_stop=10000, t_dt=0.1)*

Sets the simulation time.

Parameters:

t_start: *float, default 0*

Simulation start time (ms).

t_stop: *float, default 10000*

Simulation stop time (ms).

t_dt: *float, defulat 0.1*

Simulation time interval (ms).

genNeuronInputSignals(*signalType, period=5000., amplitude=20., offset=0., ivalue=0, heav_param= Is_heav_param*)

Generates a current signal (Isoma) intracellularly injected at the soma of the motoneuron using the built-in functions.

Parameters:

signalType: *{'Step', 'Ramp', 'sine', 'square'}*

Shape of Isoma signal.

period: *float, default 5000*

Time to the peak for the triangular 'Ramp' signal, period for the sinusoidal 'sine' signal, and period for the repetitive 'square' signal.

amplitude: *float, default 20*

Peak value in the 'Ramp', 'sine', and 'square' signal.

offset: *float, default 0*

Offset of the 'Ramp', 'sine', and 'square' signal in the y-axis.

ivalue: *float, default 0*

Initial value of the 'Ramp' signal at the simulation start.

heav_param: *list*

Parameter values of the heaviside step function for the 'Step' signal.

[i0, ip1, pon1, poff1, ip2, pon2, poff2, ip3, pon3, poff3, ip4, pon4, poff4, ip5, pon5, poff5, s] in the following equation,
Isoma = i0 + s*((heav(poff1-t)*heav(t-pon1)*ip1)
+ (heav(poff2-t)*heav(t-pon2)*ip2)
+ (heav(poff3-t)*heav(t-pon3)*ip3)
+ (heav(poff4-t)*heav(t-pon4)*ip4)
+ (heav(poff5-t)*heav(t-pon5)*ip5)).

genSynConSignals*(department, synType, signalType='Ramp', heav_param=Syn_heav_param, iValue=0., pValue=0.1, period=10000, tau=0., std_max=0., noise=False)*

Generates a synaptic conductance (SynCon) signal for the motoneuron.

Parameters:

department: *{*'Soma' and 'Dendrite'*}*
Location of synaptic input.

synType: *{'Excitatory', 'Inhibitory'}*
Type of synaptic input.

signalType: *{'Step', 'Ramp'}, default 'Ramp'*
Shape of synaptic input signal.

heav_param: *list*
Parameter values of the heaviside step function for the average 'Step' signal.
[i0, ip1, pon1, poff1, ip2, pon2, poff2, ip3, pon3, poff3, ip4, pon4, poff4, ip5, pon5, poff5, s] in the following equation,
SynCon = i0 + s*((heav(poff1-t)*heav(t-pon1)*ip1)
+ (heav(poff2-t)*heav(t-pon2)*ip2)
+ (heav(poff3-t)*heav(t-pon3)*ip3)
+ (heav(poff4-t)*heav(t-pon4)*ip4)
+ (heav(poff5-t)*heav(t-pon5)*ip5)).

iValue: *float, default 0*
Initial value of the average 'Ramp' signal at the simulation start.

pValue: *float, default 0.1*
Peak value of the average 'Ramp' signal.

period: *float, default 10000*
Time to the peak of the average 'Ramp' signal (ms).

tau: *float, default 0*
Time constant for noise generation in the average synaptic signal.

std_max: *float, default 0*
Standard deviation for noise generation in the average synaptic input signal.

noise: *bool, default False*
Indicator of noise in the average synaptic input signal.

genSpikeSignals*(signalType, t_start=100., t_stop=1400., freq=100., scale=0.)*

Generates a train of supra threshold current impulse signal (Iaxon) for axonal nerve stimulation.

Parameters:
signalType: *{'Random'}*
Shape of the Iaxon signal.

t_start: *float, default 100*
Start time of the Iaxon signal (ms).

t_stop: *float, default 1400*
End time of the Iaxon signal (ms).

freq: *float, default 100*
Frequency of the Iaxon signal (Hz).

scale: *float, default 0*
Degree of randomness in current impulse timing (ms).

genMuscleLengthSignals*(signalType, itime=-8., ftime=0., ivalue=0., fvalue=0.)*

Generates a muscle-tendon length signal (Xm).

Parameters :
signalType: *{'Isometric', 'Isokinetic', 'Random'}*
Condition of the muscle-tendon length.

itime : *float, default -8*
Start time of muscle-tendon length change for the 'Isokinetic' condition (ms).

ftime : *float, default 0*
End time of muscle-tendon length change for the 'Isokinetic' condition (ms).

ivalue : *float, default 0*
Initial muscle-tendon length for the 'Isometric' and 'Isokinetic' condition (mm).

fvalue : *float, default 0*
Final muscle-tendon length for the 'Isokinetic' condition (mm).

importNeuronInputSignals*(filePath)*

Creates a current signal intracellularly injected at the soma of the motoneuron (Isoma) from the user-defined data.

Parameter:
filePath: *str*
Path to the data file (e.g., *'../parameters/Isoma/Is_Tri.csv').*

importSynConSignals*(department, synType, filePath)*

Creates a synaptic conductance signal (SynCon) for the motoneuron from the user-defined data.

Parameters :
department: *{'Soma', 'Dendrite'}*
Location of synaptic input.

synType: *{‘Excitatory’, ‘Inhibitory’}*
Type of synaptic input.

filePath : *str*
Path to the data file (e.g., ‘../parameters/Isyn/sesyn_Tri_Noise.csv').

importSpikeSignals*(filePath, t_dt=0.1)*

Creates a supra threshold current impulse signal (Iaxon) for axonal nerve stimulation from the user-defined data.

Parameter :
filePath : *str*
Path to the data file (e.g., '../parameters/Iaxon/random_20hz.csv').

t_dt : *float, default 0.1*
Time resolution for plotting the imported signal.

importMuscleLengthSignals*(filePath)*

Create a muscle-tendon length signal (Xm) from the user-defined data.

Parameter :
filePath : *str*
Path to the data file (e.g., '../parameters/Xm/Xm_Sample.csv').

setNeuronInputSignals*(MN_range_1, MN_range_2)*

Applies the generated current signal (Isoma) for intracellular injection to the somata of motoneurons.

Parameters:
MN_range_1: *float*
The number of the first motoneuron.

MN_range_2: *float*
The number of the last motoneuron.

setSynConSignals*(department, MN_range_1, MN_range_2)*

Applies the generated synaptic conductance signal (SynCon) over the motoneurons.

Parameters:
department: *{‘Soma’, ‘Dendrite’}*
Location of synaptic input.

MN_range_1: *float*
The number of the first motoneuron.

MN_range_2: *float*
The number of the last motoneuron.

setSpikeSignals*(MF_range_1, MF_range_2)*

Applies the generated supra threshold current impulse signal (Iaxon) to the axonal nerves

connected with the muscle units.

Parameters:
MF_range_1 : *float*
The number of the first muscle-tendon unit.

MF_range_2 : *float*
The number of the last muscle-tendon unit.

setMuscleLengthSignals*(MF_range_1, MF_range_2)*

Applies the generated muscle-tendon length signal (Xm) to the muscle-tendon units.

Parameters :
MF_range_1 : *float*
The number of the first muscle-tendon unit.

MF_range_2 : *float*
The number of the last muscle-tendon unit.

plotNeuronInputSignal()

Plots the current signal (Isoma) intracellularly injected at the soma of the motoneuron.

plotSynConSignal*(department)*

Plots the synaptic conductance signal (SynCon) applied to the motoneuron.

Parameter:
department: *{'Soma', 'Dendrite'}*
Location of synaptic input.

plotSpikeSignal()

Plots the supra threshold current impulse signal (Iaxon) applied to the muscle-tendon unit.

plotMuscleLengthSignal()

Plots the muscle-tendon length singal (Xm) applied to the muscle-tendon unit.

setComputeNode*(node_list)*

Activates python parallel server at each remote computational node assigned for job execution under the cluster environment.

Parameter:
node_list: *list*
Node names (e.g., ['mupool-c01', 'mupool-c02', … , 'mupool-c##']).
Empty list for only use of the local computer or management node.

runSimulation*(num, node_list)*

Submits jobs to the job servers assigned for running the simulation.

Parameters:

num: *int or 'autodetect'*

The number of cores to use in the local computer or management node.
'autodetect' for all cores available in the local computer or the management node of the cluster system.

node_list: *list*

None for only use of the local computer or management node in the cluster system.
['computational node name'] for use of the specified computational nodes (e.g., ['mupool-c01', 'mupool-c02', … , 'mupool-c##']) in the cluster system.
'ALL' for use of all computational nodes set in the cluster system.

plotSimulResult*(scope_list)*

Displays simulation results online after simulation.

Parameter:

scope_list: *list*

Variable to plot in a separate window.
['G_esyn_dend', 'Firing_rate', 'Is', 'V_soma', '[Ca]_soma', 'E_Ca_soma', 'I_Naf_soma', 'm_Naf_soma', 'h_Naf_soma', 'I_Nap_soma', 'm_Nap_soma', 'I_Kdr_soma', 'n_Kdr_soma', 'I_Kca_soma', 'I_Can_soma', 'm_Can_soma', 'h_Can_soma', 'I_H_soma', 'm_H_soma', 'V_dend', '[Ca]_dend', 'E_Ca_dend', 'I_Cal_dend', 'm_Cal_dend', 'I_Naf_dend', 'm_Naf_dend', 'h_Naf_dend', 'I_Nap_dend', 'm_Nap_dend', 'I_Kdr_dend', 'n_Kdr_dend', 'I_Kca_dend', 'm_Kca_dend', 'I_Can_dend', 'm_Can_dend', 'h_Can_dend', 'I_H_dend', 'm_H_dend', 'I_esyn_soma', 'G_esyn_soma', 'I_isyn_soma', 'G_isyn_soma', 'I_esyn_dend', 'I_isyn_dend', 'G_isyn_dend'] for the model type of motoneuron and motor unit.
['A', 'Am', 'A_tilde', 'C1', 'C2', 'CaSP', 'CaSPB', 'CaSPT', 'CaSR', 'CaSRCS', 'F', 'MUAP', 'R', 'Spike', 'Vm', 'XCE', 'Xm'] for the model type of musclefibers and motorunit.

saveSimulationResults*(savePath, fileName)*

Saves the simulation result in a separate file for each cell and unit model.

Parameters:

savePath: *str*

Path to the folder where the files saving the simulation results are located (e.g., './results/').

filename: *str*

Common part in the file names (e.g., 'motoneuron' will produce the files named as motoneuron001.csv ~ motoneuron###.csv).

Contents in the file:

['Time','Firing_rate','Is','V_soma','[Ca]_soma','E_Ca_soma','I_Naf_soma','m_Naf_soma','h_Naf_soma','I_Nap_soma','m_Nap_soma','I_Kdr_soma','n_Kdr_soma','I_Kca_soma','I_Can_soma','m_Can_soma','h_Can_soma','I_H_soma','m_H_soma','V_dend','[Ca]_dend','E_Ca_dend','I_Cal_dend','I_Cal_dend','I_Naf_dend','m_Naf_dend','h_Naf_dend','I_Nap_dend','m_Nap_dend','I_Kdr_dend','n_Kdr_dend','I_Kca_dend','m_Kca_dend','I_Can_dend','m_Can_dend','h_Can_dend','I_H_dend','m_H_dend','I_esyn_soma','G_esyn_soma','I_isyn_soma','G_isyn_soma','I_esyn_dend','G_esyn_dend','I_isyn_dend','G_isyn_dend'] for the model type of motoneuron.

['Time','A','Am','A_tilde','C1','C2','CaSP','CaSPB','CaSPT','CaSR','CaSRCS','F','MUAP','R','Spike','Vm','XCE','Xm'] for the model type of musclefibers.

['Time','Firing_rate','MN_Spike','Is','V_soma','[Ca]_soma','E_Ca_soma','I_Naf_soma','m_Naf_soma','h_Naf_soma','I_Nap_soma','m_Nap_soma','I_Kdr_soma','n_Kdr_soma','I_Kca_soma','I_Can_soma','m_Can_soma','h_Can_soma','I_H_soma','m_H_soma','V_dend','[Ca]_dend','E_Ca_dend','I_Cal_dend','I_Cal_dend','I_Naf_dend','m_Naf_dend','h_Naf_dend','I_Nap_dend','m_Nap_dend','I_Kdr_dend','n_Kdr_dend','I_Kca_dend','m_Kca_dend','I_Can_dend','m_Can_dend','h_Can_dend','I_H_dend','m_H_dend','I_esyn_soma','G_esyn_soma','I_isyn_soma','G_isyn_soma','I_esyn_dend','G_esyn_dend','I_isyn_dend','G_isyn_dend','MF_Spike','A','Am','A_tilde','C1','C2','CaSP','CaSPB','CaSPT','CaSR','CaSRCS','F','MUAP','R','Vm','XCE','Xm'] for the model type of motorunit.

plotImportData*(dirPath, fileName, display, scope_list)*

Displays the simulation results saved in the files for offline analysis.

Parameters:

dirPath: *str*

Path to the folder where the files are saved (e.g., './results/').

filename: *str*

Common part in the fine names (e.g., 'motoneuron' in the files named as motoneuron001.csv ~ motoneuron###.csv).

display: *{'Individual', 'Combined', 'Sum'}*

'Individual' to plot the simulation data for each cell or unit on the separate figure.
'Combined' to plot the simulation data for each cell or unit on the same figure.
'Sum' to plot the summed simulation data across all cells or units.

scope_list: *list*

Variable to plot in a separate window.
['G_esyn_dend', 'Firing_rate', 'Is', 'V_soma', '[Ca]_soma', 'E_Ca_soma', 'I_Naf_soma', 'm_Naf_soma', 'h_Naf_soma', 'I_Nap_soma', 'm_Nap_soma', 'I_Kdr_soma', 'n_Kdr_soma', 'I_Kca_soma', 'I_Can_soma', 'm_Can_soma', 'h_Can_soma', 'I_H_soma', 'm_H_soma', 'V_dend', '[Ca]_dend', 'E_Ca_dend', 'I_Cal_dend', 'm_Cal_dend', 'I_Naf_dend', 'm_Naf_dend', 'h_Naf_dend', 'I_Nap_dend', 'm_Nap_dend', 'I_Kdr_dend', 'n_Kdr_dend', 'I_Kca_dend', 'm_Kca_dend', 'I_Can_dend', 'm_Can_dend', 'h_Can_dend', 'I_H_dend', 'm_H_dend', 'I_esyn_soma', 'G_esyn_soma', 'I_isyn_soma', 'G_isyn_soma', 'I_esyn_dend', 'I_isyn_dend', 'G_isyn_dend'] for the model type of motoneuron and motorunit.
['A', 'Am', 'A_tilde', 'C1', 'C2', 'CaSP', 'CaSPB', 'CaSPT', 'CaSR', 'CaSRCS', 'F', 'MUAP', 'R', 'Spike', 'Vm', 'XCE', 'Xm'] for the model type of musclefibers and motorunit.
