# Supplementary material for: Automating population construction and parallel simulation of biophysical models for neuromuscular cells: An inverse approach
Source: PLoS Comput Biol. 2026 Apr 24;22(4):e1014184. doi: 10.1371/journal.pcbi.1014184 (PMC13132451; doi:10.1371/journal.pcbi.1014184)
Supplement: S3 Text — (PDF) [file pcbi.1014184.s005.pdf]

Appendix A: Model equations and parameters

This section describes the models used for the current version of pNMS in mathematical detail. The derivation and validation of equations and parameters were fully presented in previous studies of cat spinal motoneurons (MNs) (1-3) and muscle-tendon fibers (MTs) (4, 5). The connections among the variable notations in the equations, codes, and result files were provided in Tables B and C in S1 Text. The default values of model parameters were separately specified in the parameter file for the motoneuron and muscle-tendon fiber in S1 Data.

1 MN model

1.1 System equations derived by applying Kirchhoff's current law at the soma (S) and dendrite (D) compartments in the conductance-based two-compartment model

$$C_{m,S}\frac{\mathrm{d}V_S}{\mathrm{dt}} = -G_{m,S}\left(V_S - E_{Leak,S}\right) - \tfrac{G_C}{p}(V_S - V_D) - I_{Naf,S} - I_{Kdr,S} - I_{K(Ca),S} - I_{Can,S} - I_{Nap,S} - I_{H,S} - I_{syn,S} + I_S$$

$$C_{m,D}\frac{\mathrm{d}V_D}{\mathrm{dt}} = -G_{m,D}(V_D - E_{Leak,D}) - \tfrac{G_C}{(1-p)}(V_D - V_S) - I_{Naf,D} - I_{Kdr,D} - I_{K(Ca),D} - I_{Can,D} - I_{Nap,D} - I_{H,D} - I_{Cal,D} - I_{syn,D}$$

where V and I indicate the transmembrane potential and current, respectively, with the exception of I_S, which represents the current intracellularly injected at the soma; $G_{m,S}$, $G_{m,D}$, G_C, $C_{m,D}$ and $C_{m,S}$ are the cable parameters; and E_{Leak} is the reversal potential for the leak current and set to -70 mV.

1.2 Active mechanisms in the somatic compartment

By default, the equilibrium potentials for Na^+ ($E_{Na,S}$) and K^+ ions ($E_{K,S}$) were set to 50 mV and -80 mV, whereas the equilibrium potential for Ca^{2+} ions ($E_{Ca,S}$) was set to vary dynamically in the somatic compartment.

- Intracellular calcium dynamics and calcium reversal potential

$$\frac{\mathrm{d}[Ca^{2+}]_{i,S}}{\mathrm{dt}} = f_S(-\alpha_S \times I_{Can,S} - K_{Ca,S} \times [Ca^{2+}]_{i,S})$$

$$E_{Ca,S} = \left(\frac{1000 \times R \times T}{Z_{ca} \times F}\right) \times \log\left(\frac{[Ca^{2+}]_{o,S}}{[Ca^{2+}]_{i,S}}\right) - 70$$

where $[\mathrm{Ca}^{2+}]_{\mathrm{o,S}} = 2\ \mathrm{mM},\ \mathrm{R} = 8.31441\frac{\mathrm{VC}}{\mathrm{mol}}\cdot\mathrm{k},\ \mathrm{T} = 309.15\ \mathrm{K},\ \mathrm{Z_{ca}} = 2,$ and $\mathrm{F} = 96485.309\ \mathrm{C/mol}$.

- Fast Na^+ ion current

$$I_{Naf,S} = G_{Naf,S} \times {m_{naf}}^3 \times h_{naf} \times \left(V_S - E_{Na,S}\right)$$

$$\frac{\mathrm{d}(m_{naf})}{\mathrm{dt}} = \alpha_m\left(1 - m_{naf}\right) - \beta_m \times m_{naf}$$

$$\frac{\mathrm{d}(h_{naf})}{\mathrm{dt}} = (\mathrm{h}_\infty - h_{naf})/\tau_h$$

$$\alpha_m = \frac{\alpha_{nafm1,S}(V_s - \alpha_{nafm2,S})}{e^{[-(V_s-\alpha_{nafm2,S})/\alpha_{nafm3,S}]} + \alpha_{nafm4,S}}$$

$$\beta_m = \frac{\beta_{nafm1,S}(V_s - \beta_{nafm2,S})}{e^{[(V_s-\beta_{nafm2,S})/\beta_{nafm3,S}]} + \beta_{nafm4,S}}$$

$$h_\infty = \frac{1}{1 + e^{[(V_s-\gamma_{nafh1,S})/\gamma_{nafh2,S}]}}$$

$$\tau_h = \frac{\gamma_{nafh6,S}}{e^{[(V_s-\gamma_{nafh3,S})/\gamma_{nafh4,S}]} + e^{[-(V_s-\gamma_{nafh3,S})/\gamma_{nafh5,S}]}}$$

- Delayed rectifier K^+ ion current

$$I_{Kdr,S} = G_{Kdr,S} \times {n_{kdr}}^4 \times (V_S - E_{K,S})$$

$$\frac{\mathrm{d}(n_{kdr})}{\mathrm{dt}} = (\mathrm{n}_\infty - n_{kdr})/\tau_n$$

$$n_\infty = \frac{1}{1 + e^{[-(V_s-\gamma_{kdrm1,S})/\gamma_{kdrm2,S}]}}$$

$$\tau_n = \frac{\gamma_{kdrm,6}}{e^{[(V_s-\gamma_{kdrm3,S})/\gamma_{kdrm4,S}]} + e^{[-(V_s-\gamma_{kdrm3,S})/\gamma_{kdrm5,S}]}}$$

- Ca^{2+}-dependent K^+ ion current

$$I_{K(Ca),S} = G_{K(Ca),S} \times ([Ca^{2+}]_{i,S}/([Ca^{2+}]_{i,S} + K_{d,S})) \times (V_S - E_{K,S})$$

- N-type Ca^{2+} ion current

$$I_{Can,S} = G_{Can,S} \times {m_{can}}^2 \times h_{can} \times (V_S - E_{Ca,S})$$

$$\frac{\mathrm{d}(m_{can})}{\mathrm{dt}} = (\mathrm{m}_\infty - m_{can})/\gamma_{canm3,S}$$

$$\frac{\mathrm{d}(h_{can})}{\mathrm{dt}} = (\mathrm{h}_\infty - h_{can})/\gamma_{canh3,S}$$

$$m_\infty = \frac{1}{1 + e^{[-(V_s-\gamma_{canm1,S})/\gamma_{canm2,S}]}}$$

$$h_\infty = \frac{1}{1 + e^{[(V_s-\gamma_{canh1,S})/\gamma_{canh2,S}]}}$$

- Persistent Na^+ ion current

$$I_{Nap,S} = G_{Nap,S} \times {m_{nap}}^3 \times (V_S - E_{Na,S})$$

$$\frac{\mathrm{d}(m_{nap})}{\mathrm{dt}} = \alpha_m(1 - m_{nap}) - \beta_m \times m_{nap}$$

$$\alpha_m = \frac{\alpha_{napm1,S}(V_s - \alpha_{napm2,S})}{e^{[-(V_s-\alpha_{napm2,S})/\alpha_{napm3,S}]} + \alpha_{napm4,S}}$$

$$\beta_m = \frac{\beta_{napm1,S}(V_s - \beta_{napm2,S})}{e^{[(V_s-\beta_{napm2,S})/\beta_{napm3,S}]} + \beta_{napm4,S}}$$

- Hyperpolarization-activated mixed cation (HCN) current

$$I_{H,S} = G_{H,S} \times m_h \times (V_S - E_{H,S})$$
$$\frac{\mathrm{d}(m_h)}{\mathrm{dt}} = (\mathrm{m}_\infty - m_h)/\gamma_{hm3,S}$$
$$m_\infty = \frac{1}{1 + e^{[(V_S + \gamma_{hm1,S})/\gamma_{hm2,S}]}}$$

- Synaptic currents

$$I_{syn,S} = I_{esyn,S} + I_{isyn,S}$$
$$I_{esyn,S} = G_{esyn,S} \times (V_S - E_{esyn,S})$$
$$I_{isyn,S} = G_{isyn,S} \times (V_S - E_{isyn,S})$$

where the reversal potentials for the excitatory ($E_{esyn,S}$) and inhibitory ($E_{isyn,S}$) synaptic currents were set to 0 mV and -75 mV, respectively.

Noise was added to the synaptic current using the following Ornstein–Uhlenbeck process (6):

$$\frac{\mathrm{d}G_{esyn,S}}{\mathrm{dt}} = -\frac{1}{\tau_{esyn,S}}(G_{esyn,S} - G_{esyn0,S}) + \sqrt{\frac{2\sigma_{esyn,S}^2}{\tau_{esyn,S}}} \cdot X_1$$
$$\frac{\mathrm{d}G_{isyn,S}}{\mathrm{dt}} = -\frac{1}{\tau_{isyn,S}}(G_{isyn,S} - G_{isyn0,S}) + \sqrt{\frac{2\sigma_{isyn,S}^2}{\tau_{isyn,S}}} \cdot X_2$$

where the time constant of the process for the excitatory ($\tau_{esyn,S}$) and inhibitory ($\tau_{isyn,S}$) synaptic conductance was set to 0.5 ms and 2 ms, $G_{esyn0,S}$ and $G_{isyn0,S}$ are the mean level of synaptic conductances, the standard deviation for the excitatory ($\sigma_{esyn,S}$) and inhibitory ($\sigma_{isyn,S}$) synaptic conductance was set to 0.03 and 0.06, and X_1 and X_2 represent the random Gaussian white noise produced from the standard normal distribution ($N(0, 1^2)$).

1.3 Active mechanisms in the dendritic compartment

By default, the equilibrium potentials for Na^+ ($E_{Na,D}$), K^+ ($E_{K,D}$) and Ca^{2+} ions ($E_{Ca,D}$) were set to 50 mV, -80 mV, and 60 mV in the dendritic compartment.

- Intracellular calcium dynamics and calcium reversal potential

$$\frac{\mathrm{d}[Ca^{2+}]_{i,D}}{\mathrm{dt}} = f_D(-\alpha_D \times (I_{Can,D} + I_{Cal,D}) - K_{Ca,D} \times [Ca^{2+}]_{i,D})$$
$$E_{Ca,D} = \left(\frac{1000 \times R \times T}{Z_{ca} \times F}\right) \times \log\left(\frac{[Ca^{2+}]_{o,D}}{[Ca^{2+}]_{i,D}}\right) - 70$$

where $[\mathrm{Ca}^{2+}]_{\mathrm{o,D}} = 2\ \mathrm{mM},\ \mathrm{R} = 8.31441 \frac{\mathrm{VC}}{\mathrm{mol}} \cdot \mathrm{k},\ \mathrm{T} = 309.15\ \mathrm{K},\ \mathrm{Z_{ca}} = 2,$ and $\mathrm{F} =$

96485.309 C/mol.

- Low-voltage activated L-type Ca^{2+} ($CA_v1.3$) ion current

$$I_{Cal,D} = s_{nm} \times G_{Cal,D} \times l_{cal} \times (V_D - E_{Ca,D})$$
$$\frac{\mathrm{d}(l_{cal})}{\mathrm{dt}} = (l_\infty - l_{cal})/\gamma_{calm3,D}$$
$$l_\infty = \frac{1}{1 + e^{[-(V_D - \gamma_{calm1,D})/\gamma_{calm2,D}]}}$$

where S_{nm} is a scaling factor representing the level of neuromodulatory input from the brainstem.

- Fast Na^+ ion current

$$I_{Naf,D} = G_{Naf,D} \times {m_{naf}}^3 \times h_{naf} \times (V_D - E_{Na,D})$$
$$\frac{\mathrm{d}(m_{naf})}{\mathrm{dt}} = \alpha_m(1 - m_{naf}) - \beta_m \times m_{naf}$$
$$\frac{\mathrm{d}(h_{naf})}{\mathrm{dt}} = (\mathrm{h}_\infty - h_{naf})/\tau_h$$
$$\alpha_m = \frac{\alpha_{nafm1,D}(V_D - \alpha_{nafm2,D})}{e^{[-(V_D - \alpha_{nafm2,D})/\alpha_{nafm3,D}]} + \alpha_{nafm4,D}}$$
$$\beta_m = \frac{\beta_{nafm1,D}(V_D - \beta_{nafm2,D})}{e^{[(V_D - \beta_{nafm2,D})/\beta_{nafm3,D}]} + \beta_{nafm4,D}}$$
$$h_\infty = \frac{1}{1 + e^{[(V_D - \gamma_{nafh1,D})/\gamma_{nafh2,D}]}}$$
$$\tau_h = \frac{\gamma_{nafh,6}}{e^{[(V_D - \gamma_{nafh3,D})/\gamma_{nafh4,D}]} + e^{[-(V_D - \gamma_{nafh3,D})/\gamma_{nafh5,D}]}}$$

- Delayed rectifier K^+ ion current

$$I_{Kdr,D} = G_{Kdr,D} \times {n_{kdr}}^4 \times (V_D - E_{K,D})$$
$$\frac{\mathrm{d}(n_{kdr})}{\mathrm{dt}} = (\mathrm{n}_\infty - n_{kdr})/\tau_n$$
$$n_\infty = \frac{1}{1 + e^{[-(V_D - \gamma_{kdrm1,D})/\gamma_{kdrm2,D}]}}$$
$$\tau_n = \frac{\gamma_{kdrm6,D}}{e^{[(V_D - \gamma_{kdrm3,D})/\gamma_{kdrm4,D}]} + e^{[-(V_D - \gamma_{kdrm3,D})/\gamma_{kdrm5,D}]}}$$

- Ca^{2+}-dependent K^+ ion current

$$I_{K(Ca),D} = G_{K(Ca),D} \times m_{kca} \times (V_D - E_{K,D})$$
$$\frac{\mathrm{d}(m_{kca})}{\mathrm{dt}} = (m_\infty - m_{kca})/\tau_m$$
$$m_\infty = \frac{1}{1 + (1 + \frac{\gamma_{kcam1,D}}{[Ca^{2+}]_{i,D}})^{\gamma_{kcam2,D}}}$$
$$\tau_m = \gamma_{kcam3,D}$$

- N-type Ca^{2+} ion current

$$I_{Can,D} = G_{Can,D} \times {m_{can}}^2 \times h_{can} \times (V_D - E_{Ca,D})$$
$$\frac{\mathrm{d}(m_{can})}{\mathrm{dt}} = (\mathrm{m}_\infty - m_{can})/\gamma_{canm3,D}$$
$$\frac{\mathrm{d}(h_{can})}{\mathrm{dt}} = (\mathrm{h}_\infty - h_{can})/\gamma_{canh3,D}$$
$$m_\infty = \frac{1}{1 + e^{[-(V_D - \gamma_{canm1,D})/\gamma_{canm2,D}]}}$$
$$h_\infty = \frac{1}{1 + e^{[(V_D - \gamma_{canh1,D})/\gamma_{canh2,D}]}}$$

- Persistent Na^+ ion current

$$I_{Nap,D} = G_{Nap,D} \times {m_{nap}}^3 \times (V_D - E_{Na,D})$$
$$\frac{\mathrm{d}(m_{nap})}{\mathrm{dt}} = \alpha_m(1 - m_{nap}) - \beta_m \times m_{nap}$$
$$\alpha_m = \frac{\alpha_{napm1,D}(V_D - \alpha_{napm2,D})}{e^{[-(V_D - \alpha_{napm2,D})/\alpha_{napm3,D}]} + \alpha_{napm4,D}}$$
$$\beta_m = \frac{\beta_{napm1,D}(V_D - \beta_{napm2,D})}{e^{[(V_D - \beta_{napm2,D})/\beta_{napm3,D}]} + \beta_{napm4,D}}$$

- Hyperpolarization-activated mixed cation (HCN) current

$$I_{H,D} = G_{H,D} \times m_h \times (V_D - E_{H,D})$$
$$\frac{\mathrm{d}(m_h)}{\mathrm{dt}} = (\mathrm{m}_\infty - m_h)/\gamma_{hm3,D}$$
$$m_\infty = \frac{1}{1 + e^{[(V_D + \gamma_{hm1,D})/\gamma_{hm2,D}]}}$$

- Synaptic currents

$$I_{syn,D} = I_{esyn,D} + I_{isyn,D}$$
$$I_{esyn,D} = G_{esyn,D} \times (V_D - E_{esyn,D})$$
$$I_{isyn,D} = G_{isyn,D} \times (V_D - E_{isyn,D})$$

where the reversal potentials for the excitatory ($E_{esyn,D}$) and inhibitory ($E_{isyn,D}$) synaptic currents were set to 0 mV and -75 mV, respectively.

Noise was added to the synaptic current using the following Ornstein–Uhlenbeck process (6):

$$\frac{\mathrm{d}G_{esyn,D}}{\mathrm{dt}} = -\frac{1}{\tau_{esyn,D}}(G_{esyn,D} - G_{esyn0,D}) + \sqrt{\frac{2\sigma^2_{esyn,D}}{\tau_{esyn,D}}} \cdot X_3$$

$$\frac{\mathrm{d}G_{isyn,D}}{\mathrm{dt}} = -\frac{1}{\tau_{isyn,D}}(G_{isyn,D} - G_{isyn0,D}) + \sqrt{\frac{2\sigma_{isyn,D}^2}{\tau_{isyn,D}}} \cdot X_4$$

where the time constant of the process for the excitatory ($\tau_{esyn,D}$) and inhibitory ($\tau_{isyn,D}$) synaptic conductance was set to 0.5 ms and 2 ms; $G_{esyn0,D}$ and $G_{isyn0,D}$ are the mean level of synaptic conductances, the standard deviations for the excitatory ($\sigma_{esyn,D}$) and inhibitory ($\sigma_{isyn,D}$) synaptic conductance were set to 0.03 and 0.06, and X_3 and X_4 are the random Gaussian white noise produced from the standard normal distribution ($N(0, 1^2)$).

1.4 Transduction delay time (t_{dealy}) over the axonal nerve

$t_{delay} = L_{nerve}/CV$

where L_{nerve} and CV indicate the nerve length and conduction velocity, respectively. In the current version of the pNMS, the L_{nerve} was set to 1 m and represented the peripheral nerve innervating the triceps surea muscle in humans.

2 MT model

2.1 System equations derived by applying the first-order chemical reaction to module 1, the dynamic calcium-activation relation to module 2, and the Hill mechanics to module 3

- Module 1: Transformation of the neural spikes to calcium dynamics

$$\frac{d[Ca_{SR}]}{dt} = -K1 \cdot CS_0 \cdot [Ca_{SR}] + (K1 \cdot [Ca_{SR}] + K2) \cdot [Ca_{SR}CS] - R + U$$

$$\frac{d[Ca_{SR}CS]}{dt} = K1 \cdot CS_0 \cdot [Ca_{SR}] - (K1 \cdot [Ca_{SR}] + K2) \cdot [Ca_{SR}CS]$$

$$\frac{d[Ca_{SP}]}{dt} = -K5 \cdot T_0 \cdot [Ca_{SP}] + (K5 \cdot [Ca_{SP}] + K6) \cdot [Ca_{SP}T] - K3 \cdot B_0 \cdot [Ca_{SP}] + (K3 \cdot [Ca_{SP}] + K4) \cdot [Ca_{SP}B] + R - U$$

$$\frac{d[Ca_{SP}B]}{dt} = K3 \cdot B_0 \cdot [Ca_{SP}] - (K3 \cdot [Ca_{SP}] + K4) \cdot [Ca_{SP}B]$$

$$\frac{d[Ca_{SP}T]}{dt} = K5 \cdot T_0 \cdot [Ca_{SP}] - (K5 \cdot [Ca_{SP}] + K6) \cdot [Ca_{SP}T]$$

where the following applies:

$R = [Ca_{SR}] \cdot R_{max} \cdot \sum_{i=1}^{n} \left(1 - \exp\left(-\frac{t-(t_i+t_{del})}{\tau_1}\right)\right) \cdot \exp\left(-\frac{t-(t_i+t_{del})}{\tau_2}\right)$ where t_i and t_{del} are the onset time of the ith neural spike and the delay time for the neural spike to elicit the corresponding muscle unit action potential, respectively.

$$U = U_{max} \cdot \left(\frac{[Ca_{SP}]^2 \cdot K^2}{1 + [Ca_{SP}] \cdot K + [Ca_{SP}]^2 \cdot K^2}\right)^2$$

$K5 = \varphi(X_m) \cdot K5_i$ where $\varphi(X_m) = \begin{cases} \varphi_1 \cdot X_m + \varphi_2, & for\ X_m < X_{m,0.5} \\ \varphi_3 \cdot X_m + \varphi_4, & for\ X_m \geq X_{m,0.5} \end{cases}$

$$K6 = \frac{K6_i}{1 + 5 \cdot \tilde{A}}$$

- Module 2: Transformation of the calcium dynamics to muscle activation

$$A(t) = \begin{cases} \tilde{A}^{\alpha_i}\ if\ \mathrm{A_m} = 0 \\ \tilde{A}^{\alpha(t)}\ if\ X_\mathrm{m} \geq X_{\mathrm{m},0.5}\ or\ V_\mathrm{m} \leq 0\ for\ \mathrm{A_m} \neq 0 \\ \dfrac{\tilde{A}^{\alpha(t)}}{\left(1 + \beta \cdot \varphi(X_m)\right) \cdot (1 + \gamma \cdot V_m)}\ if\ \mathrm{X_m} < X_{\mathrm{m},0.5}\ and\ \mathrm{V_m} > 0\ for\ \mathrm{A_m} \neq 0 \end{cases}$$

where the following applies:

$\frac{\mathrm{d}\tilde{A}}{\mathrm{dt}} = \frac{\tilde{A}_\infty - \tilde{A}}{\tau_{\tilde{A}}}$ and $\alpha(t) = \alpha_1 \cdot \left(1 + \tanh\left(\frac{t - \alpha_2}{\alpha_3}\right)\right) + \alpha_i$

$$\tilde{A}_\infty = 0.5 \cdot (1 + \tanh\ (\frac{\frac{[Ca_{SP}T]}{T_0} - C1}{C2}))$$

$$\frac{d[C1]}{dt} = (c1_{inf} - C1)/\tau_{c1}$$

$$\frac{d[C2]}{dt} = (c2_{inf} - C2)/\tau_{c2}$$

$$c1_{inf} = c1n1 \cdot \left(1 + \tanh\left(\frac{\frac{[Ca_{SP}T]}{T_0} - c1n2}{c1n3}\right)\right) + c1i,\ \tau_{c1} = c1n4$$

$$c2_{inf} = c2n1 \cdot \left(1 + \tanh\left(\frac{\frac{[Ca_{SP}T]}{T_0} - c2n2}{c2n3}\right)\right) + c2i,\ \tau_{c2} = c2n4$$

$$\tau_{\tilde{A}} = \frac{C3}{\cosh\left(\frac{\frac{[Ca_{SP}T]}{T_0} - C4}{2 \cdot C5}\right)}$$

- Module 3: Transformation of the muscle activation to force production

$$F = P_{0.5} \cdot K_{SE} \cdot (\Delta X_m - \Delta X_{CE})$$

where the following applies:

$$\frac{dX_{CE}}{dt} = \begin{cases} \dfrac{-b_0 \cdot (P_{0.5} \cdot g(X_m) \cdot A(t) - F)}{F + P_{0.5} \cdot a_0 \cdot g(X_m) \cdot A(t)}\ for\ F \leq P_{0.5} \cdot g(X_m) \cdot A(t) \\ \dfrac{-d_0 \cdot (P_{0.5} \cdot g(X_m) \cdot A(t) - F)}{2 \cdot P_{0.5} \cdot g(X_m) \cdot A(t) - F + P_{0.5} \cdot c_0 \cdot g(X_m) \cdot A(t)}\ for\ F > P_{0.5} \cdot g(X_m) \cdot A(t) \end{cases}$$

2.2 Inverse equations for Hill–Mashima coefficients

Given the four data points (($V_{S,1}$, $T_{S,1}$) and ($V_{S,2}$, $T_{S,2}$) for shortening and ($V_{L,1}$, $T_{L,1}$)

and ($V_{L,2}$, $T_{L,2}$) for lengthening) on the velocity–tension (V–T) curve under full excitation, the Hill–Mashima coefficients (a_0, b_0, c_0 and d_0) could be analytically determined via the following inverse equations:

$$a_0 = \frac{V_{S,1} \cdot T_{S,1} \cdot (P_{0.5} - T_{S,2}) - V_{S,2} \cdot T_{S,2} \cdot (P_{0.5} - T_{S,1})}{V_{S,2} \cdot (P_{0.5} - T_{S,1}) - V_{S,1} \cdot (P_{0.5} - T_{S,2})}$$

$$b_0 = \frac{V_{S,2} \cdot V_{S,1} \cdot (T_{S,1} - T_{S,2})}{V_{S,1} \cdot (P_{0.5} - T_{S,2}) - V_{S,2} \cdot (P_{0.5} - T_{S,1})}$$

$$c_0 = \frac{(2 \cdot V_{L,2} \cdot P_{0.5} - V_{L,2} \cdot T_{L,2}) \cdot (P_{0.5} - T_{L,1}) + (V_{L,1} \cdot T_{L,1} - 2 \cdot V_{L,1} \cdot P_{0.5}) \cdot (P_{0.5} - T_{L,2})}{V_{L,1} \cdot \{P_{0.5} - T_{L,2} - V_{L,2} \cdot (P_{0.5} - T_{L,1})\}}$$

$$d_0 = \frac{V_{L,1} \cdot V_{L,2} \cdot (T_{L,1} - T_{L,2})}{V_{L,2} \cdot (P_{0.5} - T_{L,1}) - V_{L,1} \cdot (P_{0.5} - T_{L,2})}$$

where $V_{S,1}$ and $V_{S,2}$ are the minimum and maximum shortening velocities, and $V_{L,1}$ and $V_{L,2}$ are the minimum and maximum lengthening velocities.

2.3 Length–tension properties

The length–tension relationship under full excitation was represented using an exponential function ($g(X_m)$) normalized with the peak force measured at the intermediate length ($X_{m,0.5}$) between the maximum ($X_{m,1}$) and minimal ($X_{m,0}$) physiological muscle–tendon length (X_m).

$$g(X_m) = \exp\left(-\left(\frac{X_m - g_1}{g_2}\right)^2\right) + g_3$$

2.4 Dynamic variation in the muscle-tendon length

Locomotor-like movement was generated via random variation in muscle-tendon length produced at a bandwidth ranging from 0 to 5 Hz; this bandwidth matched the changes observed in soleus length during unrestrained locomotion (7). The dynamic variation in the muscle-tendon length over time was calculated using the following two steps: random numbers were created in a white uniform 10-kHz sequence using a random number generator and then filtered with a low-pass FIR filter using a Blackman window. All length perturbations were centered on the intermediate muscle-tendon length (Xm,0.5 = -8 mm) between the physiological minimum (Xm,0 = -16 mm) and maximum (Xm,1 = 0 mm). Furthermore, the length signals that were not provided by the current version of the software could be defined by the user and imported directly from data files. The imported data file was denoised using the Savitzky-Golay filter built in the SciPy library (version 0.15.1).

2.5 Intramuscular electromyography model

The motor unit action potential (MUAP) was mathematically modeled to match the average shape of the intramuscular electromyogram (iEMG) measured for a single motor unit in human leg muscles (8). The equations representing the biphasic and triphasic forms of the MUAP were combined such that the amplitude and duration of

the MUAP were modulated with two parameters, A_{MUAP} and L_{MUAP}. The intramuscular electromyogram for all entire muscle units was calculated by summing all the MUAP signals generated by the individual muscle units recruited.

$$iEMG(t) = \sum_{i=1}^{n} A_{MUAP} \cdot \left(1 + \frac{t - t_i}{2} - 2 \cdot \left(\frac{t - t_i}{L_{MUAP}}\right)^2\right) \cdot \exp\left(-\left(\frac{t - t_i}{L_{MUAP}}\right)^2\right)$$

where t_i indicates the time when the ith muscle unit produces the motor unit action potential.

Appendix B: Inverse equations and lookup tables for the RMPs

1 MN model

The four passive CPs (τ_m, VA_{SD}^{DC}, VA_{SD}^{AC}, and VA_{DS}^{DC}) were correlated with the CI (R_N) using the following equations:

$\tau_m = 2.9*R_N + 4.5$
$VA_{SD}^{DC} = \exp(-D_{path}/\lambda_{SD}^{DC})$, where $\lambda_{SD}^{DC} = -9.32*R_N^3 + 82.36*R_N^2 + 680.18*R_N + 6.82$
$VA_{SD}^{AC} = \exp(-D_{path}/\lambda_{SD}^{AC})$, where $\lambda_{SD}^{AC} = 13.49*R_N^3 - 110.43*R_N^2 + 286.47*R_N + 188.03$
$VA_{DS}^{DC} = 1/(1-\exp(-\alpha_1/\alpha_2)+\exp((D_{path}-\alpha_1)/\alpha_2))$, where $\alpha_1 = 44.74*R_N^3 - 369.33*R_N^2 + 1050*R_N + 118.35$ and $\alpha_2 = 8.71*R_N^3 - 69.68*R_N^2 + 167.94*R_N + 111.56$

where the constants were set to capture experimental data on the distribution of τ_m and VA factors in adult cats (9).

The five passive RMPs ($G_{m,S}$, $G_{m,D}$, G_C, $C_{m,S}$, and $C_{m,D}$) were uniquely determined from the five CPs (R_N, τ_m, VA_{SD}^{DC}, VA_{SD}^{AC} and VA_{DS}^{DC}) using the following inverse equations:

$$G_{m,S} = \frac{1 - VA_{DS}{}^{DC}}{r_N \cdot (1 - VA_{SD}{}^{DC} VA_{DS}{}^{DC})}$$
$$G_{m,D} = \frac{p \cdot VA_{DS}{}^{DC} \cdot (1 - VA_{SD}{}^{DC})}{(1-p) \cdot r_N \cdot VA_{SD}{}^{DC} \cdot (1 - VA_{SD}{}^{DC} \cdot VA_{DS}{}^{DC})}$$
$$G_C = \frac{p \cdot VA_{DS}{}^{DC}}{r_N \cdot (1 - VA_{SD}{}^{DC} VA_{DS}{}^{DC})}$$
$$C_{m,D} = \frac{1}{\omega \cdot (1-p)} \sqrt{\frac{G_C{}^2}{(VA_{SD}{}^{AC})^2} - \{G_C + G_{m,D} \cdot (1-p)\}^2}$$
$$C_{m,S} = \frac{\tau_m \{p \cdot (1-p) \cdot \tau_m \cdot G_{m,S} \cdot G_{m,D} + p \cdot G_{m,S} \cdot (\tau_m \cdot G_C - C_{m,D}) + p^2 \cdot G_{m,S} \cdot G_{m,D} + (1-p) \cdot (\tau_m \cdot G_C \cdot G_{m,D} - G_C \cdot C_{m,D})\}}{p \cdot \{(1-p) \cdot (\tau_m \cdot G_{m,D} - C_{m,D}) + \tau_m \cdot G_C\}}$$

where the normalized somatic input resistance (r_N) was calculated by multiplying the somatic input resistance (R_N) with the surface area (0.316 mm^2 adopted from (2)) for the somatic compartment; the somatic-to-total surface area ratio (p) and the radian frequency of an action potential (ω) were set to 0.492 and 2π*250 rad, respectively.

Five active RMPs ($G_{Naf,S}$, f_S, $G_{Cal,D}$, $G_{K(Ca),D}$, and S_{NM}) were inversely determined from the five CPs (I_{rheo}, $t_{AHP1/2}$, PIC_{mag}, PIC_{decay}, and PIC_{amp}) respectively using the motoneuron model including the active mechanisms ($I_{Naf,S}$, $I_{Nap,S}$, $I_{Kdr,S}$, $I_{Can,S}$, $I_{K(Ca),S}$, and dynamic $[Ca^{2+}]_{i,S}$ at the soma and $I_{Cal,D}$ and constant $[Ca^{2+}]_{i,D}$ at the dendrite), as reported in a previous study (9).

The relationship between I_{rheo} and R_N was formulated as $I_{rheo}(R_N) = 29\cdot\exp[-2.48\cdot(R_N - 0.27)] + 3.5\cdot\exp[-1.82\cdot(R_N - 1.07)] + 3$ where the constants were set to capture experimental data on the distribution of I_{rheo} in adult cats (9). The I_{rheo} (nA) was matched by adjusting the $G_{Naf,S}$ under the passive dendrite condition. The following lookup table indicates the default relationship between $G_{Naf,S}$ and R_N

set in the pNMS.

Table A. Default relationship between R_N, I_{rheo}, and $G_{Naf,S}$

R_N (MΩ)	0.4	0.5	0.65	0.85	1.1	1.4	1.9	2.6	3.3	4
I_{rheo} (nA)	35.86	29.27	21.82	15.11	10.02	6.68	4.28	3.31	3.08	3.02
$G_{Naf,S}$ (mS/cm^2)	54.4	41.5	38.7	47.3	71.6	112.2	144.5	105.9	58	32.6

The relationship between $t_{AHP1/2}$ and R_N was formulated as $t_{AHP1/2}(R_N) = 21.4 \cdot R_N + 6.46$ for $R_N \leq 1.1$ MΩ and $8.6 \cdot R_N + 20.6$ for $R_N > 1.1$ MΩ where the constants were set to capture experimental data on the distribution of $t_{AHP1/2}$ in adult cats (9). The $t_{AHP1/2}$ (ms) was matched by adjusting the f_S under the passive dendrite condition. The default relationship between f_S and R_N was specified using the following lookup table.

Table B. Default relationship between R_N, $t_{AHP1/2}$, and f_S

R_N (MΩ)	0.4	0.5	0.65	0.85	1.1	1.4	1.9	2.6	3.3	4
$t_{AHP1/2}$ (ms)	15.02	17.16	20.37	24.65	30	32.64	36.94	42.96	48.98	55
f_S	0.2	0.19	0.18	0.175	0.015	0.01	0.009	0.007	0.006	0.005

The magnitude of persistent inward current (PIC) obtained under triangular voltage clamping condition at the soma was set to be similar (i.e., 22 nA) across the motoneuron population. The PIC magnitude was set to capture experimental data obtained on the ascending phase in adult cats (9). To match this property, the $G_{Cal,D}$ was adjusted as a function of R_N and D_{path} under voltage clamping condition at the passive soma. The default relations of $G_{Cal,D}$ to R_N and D_{path} were set in the following lookup table.

Table C. Default relationship between R_N, D_{path}, and $G_{Cal,D}$

D_{path} (mm) \ R_N (MΩ)	4.0	3.3	2.6	1.9	1.4	1.1	0.85	0.65	0.5	0.4
0.1	0.104	0.104	0.105	0.107	0.109	0.112	0.118	0.128	0.143	0.165
0.3	0.107	0.110	0.113	0.119	0.128	0.141	0.165	0.220	0.340	0.578
0.5	0.110	0.112	0.119	0.132	0.147	0.172	0.218	0.335	0.670	1.340
0.6	0.111	0.113	0.120	0.134	0.157	0.185	0.241	0.396	0.852	1.665
0.7	0.112	0.117	0.126	0.144	0.172	0.206	0.269	0.449	0.958	1.815
0.9	0.123	0.135	0.147	0.168	0.212	0.276	0.416	0.770	1.510	2.110
1.1	0.182	0.218	0.238	0.270	0.389	0.535	0.550	0.767	1.850	3.150

At default, the decay of persistent inward current during voltage clamping at the soma was assumed to be absent (fully bistable) over the motoneuron population. Accordingly, the $G_{K(Ca),D}$ was set to be close to zero across the R_N and D_{path}. In addition, the neuromodulatory inputs from the brainstem were assumed to be uniform (highly diffusive) across the motoneuron population. Accordingly, the S_{NM} was set to one over a physiological range of R_N.

2 MT model

Twenty-two RMPs were determined inversely from nine linear relations of $P_{0.5}$ to individual CPs: ① maximal isometric force, ② twitch dynamics, ③ length-dependent twitch amplitude, ④ dynamic calcium-force relationship, ⑤ movement-induced force degradation, ⑥ length-force property under full excitation, ⑦ velocity-force property under full excitation, ⑧ serial elastic stiffness, ⑨ muscle unit action potential, and ⑩ axonal nerve transmission. The following lookup table indicates the default relationships between the range model parameter and $P_{0.5}$ set in the pNMS. The maximum and minimum value of the range model parameter were adopted from the previous experimental studies for adult cats (4, 5).

Table D. Default relationship between $P_{0.5}$ and cell property

CP	RMP	Value									
①	$P_{0.5}$ (N)	.1	2.03	3.95	5.88	7.25	8.08	8.76	9.31	9.73	10
②	τ_1 (ms)	3	2.611	2.222	1.833	1.556	1.389	1.25	1.139	1.056	1
	τ_2 (ms)	25	22.67	20.33	18	16.33	15.33	14.5	13.83	13.33	13
③	φ_1 (mm^{-1})	.03	.025	.019	.014	.01	.007	.006	.004	.003	.002
	φ_3 (mm^{-1})	.01	.0081	.0062	.0042	.0029	.002	.0013	.0008	.0004	.0001
④	C1i	.12	.128	.136	.143	.149	.152	.155	.157	.159	.16
	C1n1	0	.0019	.0039	.0058	.0072	.0081	.0088	.0093	.0097	.01
	C1n4 (ms)	.0001	16.53	33.06	49.58	61.39	68.47	74.38	79.1	82.64	85
	C2i	.09	.1017	.1133	.125	.1333	.1383	.1425	.1458	.1483	.15
	C2n1	0	-.008	-.016,	-.023,	-.029	-0.03	-.035	-.037	-.039	-.04
	C2n4 (ms)	.0001	13.61	27.22	40.83	50.56	56.39	61.25	65.14	68.06	70
⑤	β	.5	.4203	.3406	.2608	.2039	.1697	.1413	.1185	.1014	.09
	γ	.001	.00084	.00069	.00053	.00042	.00036	.0003	.00026	.00022	.0002
⑥	g_1 (mm)	-8	-6.6	-5.2	-3.8	-2.8	-2.2	-1.7	-1.3	-1	-.8
	g_2 (mm)	22	21.03	20.06	19.08	18.39	17.97	17.63	17.35	17.14	17
⑦	a_0	.1	.0813	.0627	.044	.0307	.0227	.016	.0107	.0067	.004
	b_0 (mm/s)	24	38.78	53.56	68.33	78.89	85.22	90.5	94.72	97.89	100
	c_0	-.32	-.371	-.421	-.472	-.508	-.529	-.548	-.562	-.573	-.58
	d_0 (mm/s)	30	32.53	35.06	37.58	39.39	40.47	41.38	42.1	42.64	43
⑧	K_{SE} (mm^{-1})	0.4	0.353	0.307	0.26	0.227	0.207	0.19	0.177	0.167	0.16
⑨	A_{MUAP} (μV)	0.1	0.178	0.256	0.333	0.389	0.422	0.45	0.472	0.489	0.5
	L_{MUAP} (ms)	1	0.903	0.806	0.708	0.639	0.597	0.563	0.535	0.514	0.5
⑩	CV (m/s)	57	68.7	80.3	92	100.3	105.3	109.5	112.8	115.3	117

Notably, φ_2 and φ_4 could be analytically determined from the values of φ_1 and φ_3 in module 1 of the muscle–tendon fiber model as follows:

$\varphi_2 = 1-\varphi_1*(X_{m,0.5})$ and $\varphi_4 = 1-\varphi_3*(X_{m,0.5})$

For the length–tension relationship ($g(X_m)$) in module 3 of the muscle–tendon fiber model, g_3 was determined analytically when g_1 and g_2 were specified using the following equation:

$$g_3 = \exp\left(-\left(\frac{X_{m,0.5} - g_1}{g_2}\right)^2\right) - 1$$

3 User-defined distribution of the range model parameters

In pNMS, the heterogeneity of the population model was determined by continuously varying the intrinsic cellular properties represented by the range model parameters (Tables 1 and 2). The range model parameters were varied as a function of R_N in the motoneuron population, $P_{0.5}$ in the muscle-tendon fiber population, and R_N and $P_{0.5}$ in the motor unit population model. At default, all range model parameters were determined from the inverse equations and lookup tables embedded in the code (simulation_engine.py in S1 Data) to represent the relationships between the range model parameter and the cell indicator (R_N for the motoneuron and motor unit and $P_{0.5}$ for the muscle-tendon fiber) observed in normal adult cats. For forward modeling, the range model parameter–cell indicator relationship could be reset in a linear and nonlinear manner using the built-in functions or by importing the data file in a plain text format (i.e., CSV format). The relationships between cell indicator and model number, R_N and D_{path}, and R_N and $P_{0.5}$ could also be set using the built-in functions or by importing the data file in a linear and nonlinear form.

The built-in function for the linear relationship was implemented using the first-order polynomial function:

$$y = a_1 * x + a_0$$

where x and y indicate the model number and the cell indicator, the cell indicator and the range model parameter, or R_N and $P_{0.5}$.

The four types of nonlinear functions were built in to represent increasing concave and convex curves and decreasing concave and convex curves. The built-in function for the nonlinear relationship was implemented by mapping the relationships (i.e., cell indicator–model number, range model parameter–cell indicator, D_{path}–R_N, and $P_{0.5}$–R_N) using the following exponential function:

$$y = \begin{cases} \begin{cases} -\exp(-x) \text{ for increasing concave shape} \\ -\exp(x) \text{ for decreasing concave shape} \end{cases} \\ \begin{cases} \exp(x) \text{ for increasing convex shape} \\ \exp(-x) \text{ for decreasing convex shape} \end{cases} \end{cases}$$

where the curvature of the nonlinear relationship is determined in accordance with the range of x values that induce the desired shape of the exponential curve; the default range of range model parameter, R_N, D_{path}, and $P_{0.5}$, set within the code (simulation_engine.py in S1 Data) based on the cat experimental data, can be flexibly reset when the built-in function (setParameters in Table 3) is specified.

Appendix C: Considerations for software implementation

1 Simulation considerations

1.1 Topology and geometry

For MNs, the electrotonic structure of the dendrites was mapped on the two-compartment MN model by capturing the three voltage attenuation factors (VA_{SD}^{DC}, VA_{DS}^{DC}, and VA_{SD}^{AC}) characterized between the soma and all dendritic sites at a similar path length (D_{path}) from the soma. The equivalent input resistance ($R_{N,D}$) for the dendritic sites at a similar D_{path} was implicitly incorporated to the dendritic compartment by the formulation of the R_N multiplied by the asymmetry ratio ($VA_{SD}^{DC}/VA_{DS}^{DC}$) of the dendritic signal propagation, as reported in a previous study (3, 9).

Regarding MTs, the force produced by individual muscle fibers was assumed to be scalable from that produced by the sarcomere and that the muscle fibers were uniformly arranged with identical lengths within the muscle (10). The number and cross-sectional area of muscle fibers innervated by a single motoneuron were reflected in the force produced during isometric contractions at the intermediate muscle-tendon length ($X_{m,\ 0.5}$) between its physiological maximum ($X_{m,\ 1}$) and minimum ($X_{m,\ 0}$) under full excitation (11).

1.2 Biophysical properties

Regarding MN model, the passive dynamics of the membrane potential were captured using the five cable parameters (i.e., $G_{m,S}$, $G_{m,D}$, $C_{m,S}$, $C_{m,D}$, and G_C). The rhythmic firing activity of motoneurons was produced at the somatic compartment via interactions between the following six active currents (i.e., $I_{Na,f}$, $I_{K,dr}$, $I_{Ca,N}$, $I_{K(Ca)}$, $I_{Na,p}$, and I_H). The plateau potentials and voltage oscillations at the dendritic compartment were produced using the following seven active currents (i.e., $I_{Na,f}$, $I_{K,dr}$, $I_{Ca,N}$, $I_{K(Ca)}$, $I_{Na,p}$, I_H, and $I_{Ca,L}$). The dynamics of the intracellular calcium concentration was also considered to update the $I_{K(Ca)}$ (12) and equilibrium potential (E_{Ca}) for calcium ions (13) at both the somatic and dendritic compartments. The Hodgkin-Huxley-type formulation was applied for modeling the voltage-gated ion currents (i.e., $I_{Na,f}$, $I_{K,dr}$, $I_{Ca,N}$, $I_{Na,p}$, $I_{Ca,L}$, and I_H) for both compartments. The subcellular parameters (i.e., f, α, K_{Ca}, and K_d) were added to reflect the calcium fluctuations that are measurable over the soma and dendritic areas using calcium imaging techniques (14).

For MT model, the first module (module 1) represented the transformation of the spike signals from the motoneurons into the concentration dynamics of calcium (Ca) and Ca bound to troponin (CaT) in the sarcoplasm (SP) (15-17). The sarcoplasmic Ca concentration was determined by the release (R) and reuptake (U) of Ca through the membrane of a sarcoplasmic reticulum (SR) containing free Ca and calsequestrin (CS) and the interactions with free Ca-buffering proteins (B) and troponins (T) in the SP. The second module (module 2) addressed the conversion of the CaT concentration in the SP to the level of muscle activation (A(t)) (18-20). This conversion was based on the nonlinear (i.e., sigmoidal) relationship between the Ca concentration in the SP and force production under steady-state conditions,

reflecting the cooperativity in cross-bridge formation (21). The dynamics of A(t) was further modulated as a function of the muscle-tendon length (X_m) and velocity (V_m) to reflect the degradation of muscle activation during dynamic variation in the whole muscle length (22). The CaT-A relationship was dynamically changed to capture the progressive force decline (or sag phenomenon) during unfused isometric contractions (4). The third module (module 3) simulated the transformation of A(t) into the muscle force based on Hill-type mechanics consisting of the contractile element (CE) and serial elastic element (SE) lumping intracellular (i.e., connective tissues and filaments) and extracellular (i.e., tendon and aponeurosis) muscle compliance (23, 24) or a whole muscle preparation (22, 25). The muscle unit action potential (MUAP) measured intramuscularly was mathematically modeled by combining previously reported formulations for the biphasic and triphasic MUAP forms, and its amplitude and duration were adjusted by two parameters (i.e., A_{MUAP} and L_{MUAP}) (26) (27).

In terms of peripheral nerves for MU model, the diameter of a single axonal nerve rooted from a spinal motoneuron was reflected in the conduction velocity (CV) of the neural signals reaching the muscle fibers (i.e., muscle unit) that the motoneuron innervates (28). The endings of a single axonal nerve were assumed to be uniformly distributed over individual muscle fibers comprising a muscle unit (29) and perfectly induced one-to-one excitation-contraction coupling between a motoneuron and muscle unit (30).

For population model, the current version of pNMS enables two modes (homogeneous and heterogeneous) of population modeling and simulations for the spinal motoneurons, skeletal muscle fibers, and motor units, respectively. In the homogeneous mode, all model parameters are initialized with default values set in a model parameter file and applied identically to all cell models consisting of the homogeneous population model. In the heterogeneous mode, the cell-specific properties are assigned to individual cell models by systematically varying the relevant model parameters referred to as the range model parameters in this study.

1.3 Input conditions

In this study, for spinal motoneurons, intracellular stimulation and synaptic inputs were considered. Two types of intracellular stimulation (i.e., I_{soma}) protocols were applied at the soma (31, 32): (1) a long-lasting current step along with alternating excitatory and inhibitory current pulses to test the cell excitability at the steady state and (2) a current ramp that slowly rises and falls to simulate the input–output relationship at a wide range of stimulation intensities. Additionally, periodic square and sine wave functions are added for further analysis with different types of inputs from other neurons (33). Regarding the synaptic inputs, both excitatory (i.e., I_{esyn}) and inhibitory (i.e., I_{isyn}) ionotropic synaptic inputs were applied at the soma and dendrites. Similar to the case for intracellular stimulation, the synaptic conductance was varied in two forms: a long-lasting step along with alternating pulses and an ascending and descending triangular shape over time. Noise was selectively incorporated into the mean synaptic conductance to reflect noisy background activity and synaptic transmission (34). For realistic simulations, the amplitude of the synaptic noise was modulated in proportion to the magnitude of the mean synaptic

conductance (35). In addition, the metabotropic synaptic inputs (i.e., NM) mediated by monoaminergic brainstem neuromodulation were reflected by scaling the peak conductance ($G_{Ca,L}$) of L-type calcium channels clustered over the dendrites (36). The level of brainstem neuromodulation was adjusted by multiplying $G_{Ca,L}$ with the range model parameter of S_{NM}. Notably, input signals that were not provided by the current version of the software could be imported directly from the data file defined by the user.

The muscle unit is excited through stimulating an axonal nerve with a suprathreshold current impulse (i.e., I_{axon}) sufficient to evoke a spike over the axon at both regular (or constant) and irregular (or random) frequencies (30). The current impulses at an irregular frequency were produced from a normal (Gaussian) distribution for a given mean frequency and standard deviation. The delay of signal transmission over the axonal nerve was reflected by varying its conduction velocity. Direct stimulation over the muscle belly was also considered by setting the axonal conduction velocity to be the same for all muscle units. Furthermore, users could directly import the data file for input signals not provided by the current software version.

The muscle-tendon length (i.e., X_m) was changed over the full physiological range from the minimum ($X_{m,0}$) of −16 mm to the maximum ($X_{m,1}$) of 0 mm along with the intermediate length ($X_{m,0.5}$) of −8 mm that was identified from cat hindlimb muscles (22). In this study, the muscle-tendon length varied in three modes: (1) a constant length under steady-state conditions (isometric), (2) a constant length change over time (isokinetic) and (3) locomotor-like movement (dynamic). Under dynamic conditions, locomotor-like movement was generated via random variation in the muscle-tendon length produced at bandwidths ranging from 0 to 5 Hz; this bandwidth range matches the changes observed in soleus length during unrestrained locomotion of adult cats (7). All length perturbations were centered on an operating point of −8 mm less than the physiological maximum length (0 mm). Furthermore, length signals that were not provided by the current version of the software could be defined by the user importing user-defined data files.

1.4 Numerical integration

The accuracy, stability, and performance of the computer simulation were considered in selecting the optimal method for the numerical integration of the computational models for spinal motoneurons, muscle-tendon fibers, and motor units. Among the ordinary differential equation (ODE) solvers available in the Python library (i.e., scipy), LSODA with the optimized max_step (i.e., 10 times simulation temporal resolution) was the best option for the motoneuron and muscle-tendon fiber models. Although the simulation with VODE was faster than that with LSODA for both the motoneuron and muscle-tendon fiber models, the VODE solver caused an unstable simulation and inaccurate integration in both the motoneuron and muscle-tendon fiber models. For the motoneuron population model, changing the simulation time caused unstable simulations with the VODE solver. The muscle-tendon fiber population model could not reliably respond to the first current pulse injection with the VODE solver. However, VODE was used for the motoneuron part in the motor unit model because the same numerical solvers were not allowed to be used at the same time in the current version of the Python environment. The simulations using

other methods (i.e., dopri5 and dop853) were much slower for all three models (i.e., motoneuron, muscle-tendon fiber, and motor unit). For the stochastic simulations, including the noise in the synaptic conductance, the LSODA was also applied to numerically solve the motoneuron, muscle-tendon fiber, and motor unit models.

2 Software implementation

2.1 Extensibility

An object-oriented programming paradigm was employed to improve not only the extensibility but also the maintenance and reusability of the software components (37). All components related to the input, model, simulation and output were independently designed in the form of a class. The unit and population models were hierarchically constructed by combining relevant classes. At the cellular level, the motoneuron and muscle-tendon fiber models were implemented as a child class inherited from a parent class that defined the common features shared between cell models. Similarly, at the unit level, the motor unit model was implemented as a child class composed of the motoneuron and muscle-tendon fiber classes. For the population (or pool) level, the motoneuron, muscle-tendon fiber, and motor unit population model were implemented as child classes composed of motoneurons, muscle-tendon fibers, and motor unit classes, respectively. The functions used to generate the distribution of range model parameter values over the heterogeneous population models were encapsulated in an independent class. The generation of the input signals was implemented as an association class that could be referred in the population class. Parallel simulation and result management were also implemented as an association class that could be referred in the population class. The structural and dynamical aspects of the software were designed using the unified modeling language (UML 2.1 standard, www.omg.org/spec/UML/2.1.2).

2.2 Interoperability

To ensure interoperability between the operating systems and platforms, the current version of pMPUS software was implemented with the pure Python (version 2.7) language and developed in the Anaconda software environment (version 2.2.0). The Python packages previously developed were used to manage the data (Pandas version 0.15.2), solve the models (Scipy version 0.15.1), plot the results (Matplotlib version 1.4.3 and PyQt4 version 4.11.4), and execute the parallel computation (pp version 1.6.5).

2.3 Parallelism

Fig 3 shows the components and their input-output signals considered in the current version of pNMS. pNMS was designed to simulate the behaviors of individual cells or unit models consisting of the population models under the parallel computing environment (Fig 2). The parallel simulation was performed through the parallel python module (i.e., pp, https://www.parallelpython.com/). This module was chosen because of the easy implementation of the parallel computing environment along with various useful services, including dynamic processor allocation, dynamic load

balancing, and fault tolerance management. The pp module allows parallel computation across multiple computer systems, including a multi-core computer, high-performance cluster system, and computer network through the internet.

2.4 Packaging

The software modules of pNMS were packed into two packages for the simulation engine and the application programming interface. The simulation engine package includes all classes, and the application programming interface package contains command-line (or API) functions that help the users program virtual experiments of the neuromuscular system via the pNMS simulation engine. The contents of these packages are fully provided in Table A in S1 Text and Table 3. The source codes built into the current version of the software are presented in S1 Data.

Appendix D: Instructions for parallel simulation

1 Multicore computer environment

The exemplary simulations presented in this study were conducted on a local computer with eight logical cores. The parallel simulation in a multicore computer can be performed via the following four steps.

1) Construction of the Python-based parallel computing environment
The relevant Python version and libraries are installed to run simulations using the pNMS software.

2) Installation of the pNMS software
The folder (i.e., pNMS), including the pNMS packages (application_programming.py and simulation_engine.py) and the shell file (ppserver.sh) for ppserver activation, is moved into the folder that contains the application code and the data folder, including the user-defined files for model parameters and input signals.

3) Specification of the computational node
As in the example codes (S2 Data), the code of node_list is set to [ ] to use only a local computer. The number of cores (NC) to be used is specified by an integer or 'autodetect' for all available cores in the code of cf.runSimulation(NC, None). An example code is as follows:

```
node_list = []
cf.setComputeNode(node_list)
cf.runSimulation('autodetect',None)
```

4) Running of the application code
The application code can be run in any Python development environment or command window.

2 High-performance computing environment

Exemplary simulations conducted for this study using pNMS can be performed in a high-performance computing environment consisting of multiple network computers through the following five steps.

1) Construction of the Python-based parallel computing environment
The venv module is installed in the shared folder through the network file system (NFS) in the management node. Within the venv folder, the relevant Python version and libraries are installed to run simulations using the pNMS software.

2) Installation of the pNMS software
The folder (i.e., pNMS), including the pNMS packages (application_programming.py and simulation_engine.py) and the shell file (ppserver.sh) for activation of venv and ppserver on individual computational nodes, is moved to the Python library folder (e.g., /venv/lib/python2.6/site-packages) within the venv folder. The example code of

ppserver.sh running on all the participating computational nodes is as follows:

```
source simdata/project/2024/venv/bin/activate
ppserver.py -a -p 60000 -t 10
```

3) Specification of the computational nodes in the application code
In the example codes (S2 Data), the code of node_list = [ ] for a local computer first needs to be changed to the form of node_list = ['mupool-c01', 'mupool-c02'], where 'mupool-c01' and 'mupool-c02' are example names of computational nodes participating in parallel simulation. The computational nodes need to be set using the API function of cf.setComputeNode(node_list). Finally, the code of cf.runSimulation(16, None), which uses only the management node (16 cores and 1 CPU) without computational nodes, needs to be modified to cf.runSimulation(0, 'ALL'), which uses all computational nodes set in the cluster system. An example code is as follows:

```
node_list = ['mupool-c01', 'mupool-c02']
cf.setComputeNode(node_list)
cf.runSimulation(0, ‘ALL’)
```

4) Movement of the application code and the data folder
The application code and the data folder, including the user-defined files for model parameters and input signals, are moved to the folder where the venv module is installed.

5) Running of the application code in the management node
The venv module needs to be activated first in the management node, as shown in the following example.

```
[root@mupool-m ~]# cd /simdata/project/2024
[root@mupool-m 2024]# source venv/bin/activate
(venv)[root@mupool-m 2024]#
```

Then, the directory needs to be changed to the folder containing the application file, which can be executed using the python command, as shown in the following example.

```
(venv)[root@mupool-m 2024]# cd pNMS
(venv)[root@mupool-m pNMS]# python fig_S3.py
```

References

1. Kim H, Jones KE. The retrograde frequency response of passive dendritic trees constrains the nonlinear firing behaviour of a reduced neuron model. PloS one. 2012;7(8):e43654.
2. Kim H, Jones KE, Heckman CJ. Asymmetry in signal propagation between the soma and dendrites plays a key role in determining dendritic excitability in motoneurons. PloS one. 2014;9(8):e95454.
3. Kim H, Major LA, Jones KE. Derivation of cable parameters for a reduced model that retains asymmetric voltage attenuation of reconstructed spinal motor neuron dendrites. Journal of computational neuroscience. 2009;27(3):321-36.
4. Kim H, Heckman CJ. A dynamic calcium-force relationship model for sag behavior in fast skeletal muscle. PLoS Comput Biol. 2023;19(6):e1011178.
5. Kim H, Sandercock TG, Heckman CJ. An action potential-driven model of soleus muscle activation dynamics for locomotor-like movements. J Neural Eng. 2015;12(4):046025.
6. Destexhe A, Rudolph M, Fellous JM, Sejnowski TJ. Fluctuating synaptic conductances recreate in vivo-like activity in neocortical neurons. Neuroscience. 2001;107(1):13-24.
7. Goslow GE, Jr., Reinking RM, Stuart DG. The cat step cycle: hind limb joint angles and muscle lengths during unrestrained locomotion. J Morphol. 1973;141(1):1-41.
8. Merletti R, Farina D. Analysis of intramuscular electromyogram signals. Philos Trans A Math Phys Eng Sci. 2009;367(1887):357-68.
9. Kim H. Impact of the localization of dendritic calcium persistent inward current on the input-output properties of spinal motoneuron pool: a computational study. Journal of applied physiology. 2017;123(5):1166-87.
10. Winters TM, Takahashi M, Lieber RL, Ward SR. Whole muscle length-tension relationships are accurately modeled as scaled sarcomeres in rabbit hindlimb muscles. J Biomech. 2011;44(1):109-15.
11. Conwit RA, Stashuk D, Tracy B, McHugh M, Brown WF, Metter EJ. The relationship of motor unit size, firing rate and force. Clinical neurophysiology : official journal of the International Federation of Clinical Neurophysiology. 1999;110(7):1270-5.
12. Booth V, Rinzel J, Kiehn O. Compartmental model of vertebrate motoneurons for Ca2+-dependent spiking and plateau potentials under pharmacological treatment. J Neurophysiol. 1997;78(6):3371-85.
13. McIntyre CC, Grill WM. Extracellular stimulation of central neurons: influence of stimulus waveform and frequency on neuronal output. J Neurophysiol. 2002;88(4):1592-604.
14. Larkum ME, Rioult MG, Luscher HR. Propagation of action potentials in the dendrites of neurons from rat spinal cord slice cultures. J Neurophysiol. 1996;75(1):154-70.
15. Westerblad H, Allen DG. The role of sarcoplasmic reticulum in relaxation of mouse muscle; effects of 2,5-di(tert-butyl)-1,4-benzohydroquinone. J Physiol. 1994;474(2):291-301.
16. Stephenson DG, Wendt IR. Length dependence of changes in sarcoplasmic calcium concentration and myofibrillar calcium sensitivity in striated muscle fibres. J Muscle Res Cell Motil. 1984;5(3):243-72.

17. Baylor SM, Hollingworth S. Intracellular calcium movements during excitation-contraction coupling in mammalian slow-twitch and fast-twitch muscle fibers. J Gen Physiol. 2012;139(4):261-72.
18. Stephenson DG, Williams DA. Calcium-activated force responses in fast- and slow-twitch skinned muscle fibres of the rat at different temperatures. J Physiol. 1981;317:281-302.
19. Metzger JM, Moss RL. Calcium-sensitive cross-bridge transitions in mammalian fast and slow skeletal muscle fibers. Science. 1990;247(4946):1088-90.
20. Brandt PW, Cox RN, Kawai M. Can the binding of Ca2+ to two regulatory sites on troponin C determine the steep pCa/tension relationship of skeletal muscle? Proc Natl Acad Sci U S A. 1980;77(8):4717-20.
21. Shames DM, Baker AJ, Weiner MW, Camacho SA. Ca(2+)-force relationship of frog skeletal muscle: a dynamic model for parameter estimation. Am J Physiol. 1996;271(6 Pt 1):C2062-71.
22. Sandercock TG, Heckman CJ. Force from cat soleus muscle during imposed locomotor-like movements: experimental data versus Hill-type model predictions. J Neurophysiol. 1997;77(3):1538-52.
23. Gordon AM, Huxley AF, Julian FJ. The variation in isometric tension with sarcomere length in vertebrate muscle fibres. J Physiol. 1966;184(1):170-92.
24. Bottinelli R, Canepari M, Pellegrino MA, Reggiani C. Force-velocity properties of human skeletal muscle fibres: myosin heavy chain isoform and temperature dependence. J Physiol. 1996;495 (Pt 2)(Pt 2):573-86.
25. Brown IE, Cheng EJ, Loeb GE. Measured and modeled properties of mammalian skeletal muscle. II. The effects of stimulus frequency on force-length and force-velocity relationships. J Muscle Res Cell Motil. 1999;20(7):627-43.
26. Cisi RR, Kohn AF. Simulation system of spinal cord motor nuclei and associated nerves and muscles, in a Web-based architecture. Journal of computational neuroscience. 2008;25(3):520-42.
27. Aminoff MJ. Aminoff's electrodiagnosis in clinical neurology. [S. l.]: Saunders; 2012. Available from: https://www.clinicalkey.com/dura/browse/bookChapter/3-s2.0-C20100655994.
28. Cullheim S, Ulfhake B. Relations between cell body size, axon diameter and axon conduction velocity of triceps surae alpha montoneurons during the postnatal development in the cat. J Comp Neurol. 1979;188(4):679-86.
29. Rodriguez Cruz PM, Cossins J, Beeson D, Vincent A. The Neuromuscular Junction in Health and Disease: Molecular Mechanisms Governing Synaptic Formation and Homeostasis. Front Mol Neurosci. 2020;13:610964.
30. Rack PM, Westbury DR. The effects of length and stimulus rate on tension in the isometric cat soleus muscle. J Physiol. 1969;204(2):443-60.
31. Hounsgaard J, Hultborn H, Jespersen B, Kiehn O. Bistability of alpha-motoneurones in the decerebrate cat and in the acute spinal cat after intravenous 5-hydroxytryptophan. J Physiol. 1988;405:345-67.
32. Lee RH, Heckman CJ. Bistability in spinal motoneurons in vivo: systematic variations in rhythmic firing patterns. J Neurophysiol. 1998;80(2):572-82.
33. Baker SN. Oscillatory interactions between sensorimotor cortex and the periphery. Curr Opin Neurobiol. 2007;17(6):649-55.
34. Destexhe A, Pare D. Impact of network activity on the integrative properties of neocortical pyramidal neurons in vivo. J Neurophysiol. 1999;81(4):1531-47.
35. Powers RK, Elbasiouny SM, Rymer WZ, Heckman CJ. Contribution of

intrinsic properties and synaptic inputs to motoneuron discharge patterns: a simulation study. J Neurophysiol. 2012;107(3):808-23.
36. Lee RH, Heckman CJ. Adjustable amplification of synaptic input in the dendrites of spinal motoneurons in vivo. J Neurosci. 2000;20(17):6734-40.
37. Bal H, Grune D. Programming language essentials: Addison-Wesley Longman Publishing Co., Inc.; 1994.
