# Supplementary material for: Automating population construction and parallel simulation of biophysical models for neuromuscular cells: An inverse approach
Source: PLoS Comput Biol. 2026 Apr 24;22(4):e1014184. doi: 10.1371/journal.pcbi.1014184 (PMC13132451; doi:10.1371/journal.pcbi.1014184)
Supplement: Attachment — Submitted filename: replies_rev1_f_Clean.pdf [file pcbi.1014184.s007.pdf]

February 28, 2026

The Editorial Office
PLOS Computational Biology

Reply to Reviewer Comments

RE: Revision of the manuscript "Automating population construction and parallel simulation of biophysical models for neuromuscular cells: An inverse approach"

Thank you for the opportunity to resubmit the revision of our manuscript and reply to the concerns raised by the Reviewers. We believe the process of review and revision has created a much stronger manuscript that will communicate our novel findings to the readership of NeuroImage. Our point-by-point responses (purple) to the Reviewers' comments (black) are given below with line numbers. We have taken the Reviewers' concerns seriously and made significant changes to the original manuscript.

Reviewers' comments

Reviewer #1:
General Overview
This manuscript presents pNMS, a Python framework for building and simulating populations of motoneurons (MNs), motor units (MUs), and muscle-tendon fibers (MTs) with heterogeneous properties. Overall, the work is solid and clearly represents a useful tool for the neuromuscular modeling community. I appreciate the combination of open source, object-oriented design, efficient parallel computing, and detailed inclusion of cellular features. The manuscript is well-written, logically structured, and provides enough methodological detail to reproduce the simulations, which makes it very accessible for other groups.

Novelty and Strengths
pNMS stands out for its use of inverse modeling to automatically set population parameters based on measurable cellular properties. This makes it possible to build heterogeneous MN–MT–MU populations in a systematic way. The model also captures detailed cellular-to-population dynamics, and its parallelized simulation on multicore systems or clusters makes it practical for larger-scale studies. Finally, being open-source and extensible in Python means it can be adapted, integrated, or expanded by other researchers, which is a big plus.

Reply: We appreciate the reviewer for critical and constructive comments and recommendations on the manuscript.

Major Comments
1. MUAP generation and fiber synchrony
Generating MUAPs for each individual fiber is a great feature, it lets you see how fiber-level differences affect the overall MUAP. That said, the current setup seems to

assume all fibers in a MU fire synchronously. This could limit its usefulness for conditions where fibers don’t activate perfectly together, like fatigue, sarcopenia, or NMJ dysfunction. It would be good for the authors to discuss this limitation and maybe hint at ways it could be handled in future versions.

Reply: We appreciate the reviewer bringing this critical issue to our attention. The reviewer is correct. All muscle fibers in a single MU were assumed to activate synchronously in the present study. However, the fibers within an MU can be activated asynchronously under abnormal conditions, such as fatigue, sarcopenia, or neuromuscular junction dysfunction. One way to simulate the asynchronous activation of muscle fibers in an MU in the current pNMS is to construct an MU population comprising identical MNs (i.e., same R_N) and MTs (i.e., same $P_{0.5}$) except for nerve conduction velocity (i.e., varying CV). Assigning different CV values by importing $P_{0.5}$–CV correlation data (Appendix B in Supplemental Information 5) can result in different time delays in the MUAP and force responses of individual MTs to the motoneuron spike in the pNMS. An example simulation was presented in Fig. S9 (Supplemental Information 1).

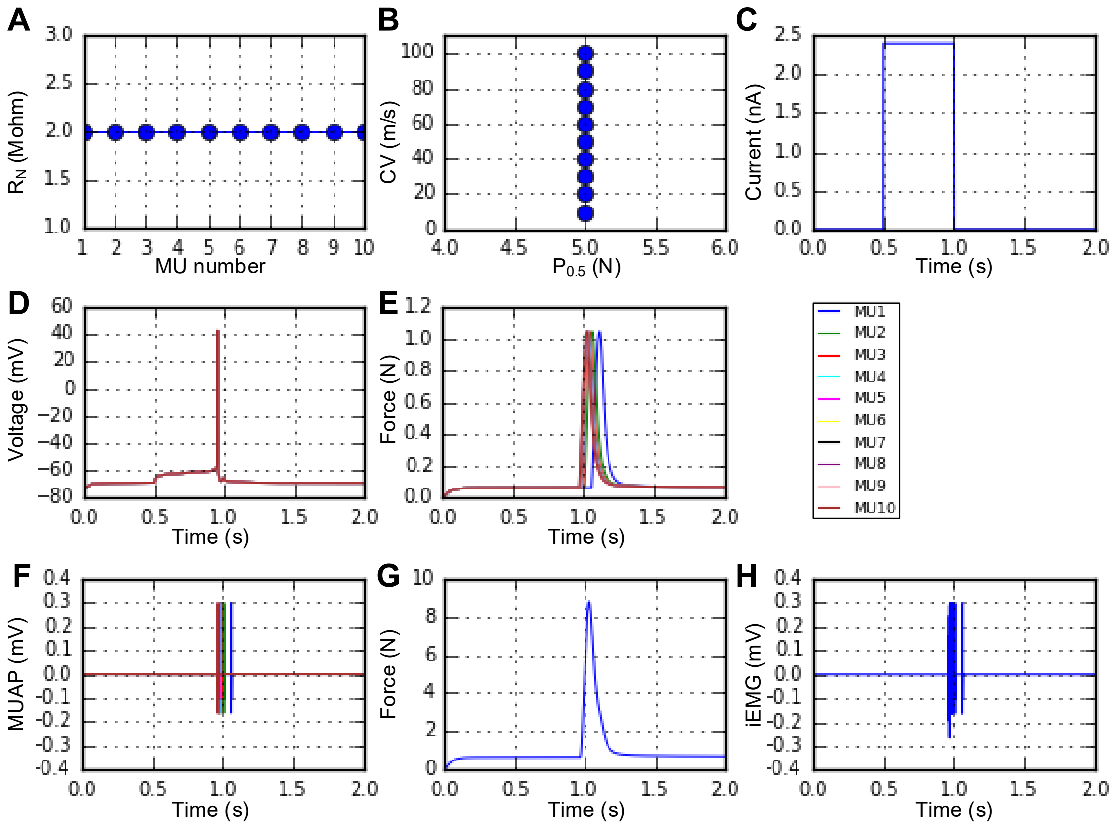

Figure S9. Simulation of asynchronous activation of muscle fibers in a motor unit. A. Motoneuron input resistance (R_N) across the motor unit population. **B.** Axonal conduction velocity across the motor unit population. **C.** Intracellular input to the somata of the motoneurons. **D.** Action potentials of the motoneurons. **E.** Force responses of individual muscle fibers. **F.** MUAPs of individual muscle fibers. **G.** Force production by the entire motor unit population. **H.** Intracellular EMG (iEMG) produced by the entire motor unit population.

We have clearly stated this limitation and how to handle it in the current pNMS version in the revised manuscript's Discussion (Line 1030-1039).

2. Heterogeneous fiber composition within a MU
The manuscript focuses on homogeneous fiber types within a MU, but it seems the framework could, in principle, mix fiber types. It would be helpful if the authors commented on how feasible this actually is, and whether there are limits, like computational cost, or issues with the inverse modeling framework if you combine fiber types without well characterized CP correlations.

Reply: Thanks for your comments. This study also assumed that the same type of muscle fibers comprise an MU. However, individual muscle fibers within an MU may exhibit different mechanical properties. This scenario can be simulated by constructing an MU population comprising identical MNs (i.e., same R_N) but heterogeneous MTs (i.e., varying $P_{0.5}$). The R_N–$P_{0.5}$ correlation can be updated by importing user-specified data (Appendix B in Supplemental Information 5). The figure below shows a simulation example (Figure S10 in Supplemental Information 4).

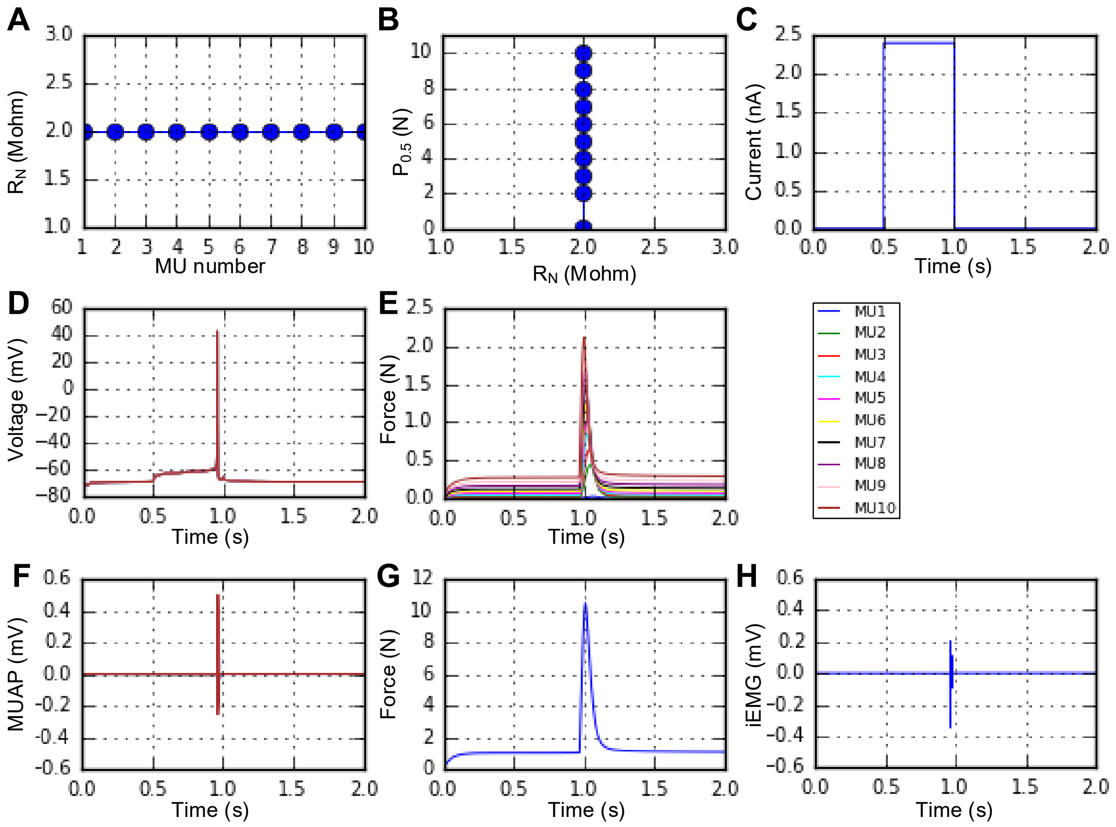

Figure S10. Simulation of a motor unit with heterogeneous muscle fibers. A. Motoneuron input resistance (R_N) across the motor unit population. **B.** Muscle fiber peak force ($P_{0.5}$) across the motor unit population. **C.** Intracellular input to the somata of the motoneurons. **D.** Action potentials of the motoneurons. **E.** Force responses of individual muscle fibers. **F.** MUAPs of individual muscle fibers. **G.** Force production by the entire motor unit population. **H.** Intracellular EMG (iEMG) produced by the entire motor unit population.

Ultimately, however, the current MU model, which lumps all MTs into a single MT model, needs to be expanded to more realistically and precisely account for MT heterogeneity within an MU. To alert the readers to this issue raised by the reviewer, we have added the sentences to the Discussion in the revised manuscript (Line 1041-1049).

3. Stimulus protocol and translation to voluntary contractions
The validation is based on stimuli applied to anesthetized cats, so non-voluntary activation. It would be useful for the authors to clarify that using inverse modeling to predict voluntary contractions isn't straightforward. This doesn't have to be a flaw, but highlighting the intended scope of the model would help readers.

Reply: We agree with the reviewer that The inverse approach proposed in this study has been tested primarily for anesthetized, nonvoluntary cats. The reason was the lack of available data obtained during voluntary contractions. Thus, the current version of pNMS has an inherent limitation in predicting voluntary behaviors. However, the current pNMS software may serve as an efficient tool for systematically investigating the input-output properties of the neuromuscular system at single-cell resolution. Moreover, future studies will validate the current pNMS software using empirical data obtained during voluntary control.

To clarify this issue, we have added the sentences to the Discussion in the revised manuscript (Line 1051-1058).

4. Validation with experimental data and inverse modeling
The paper emphasizes that inverse modeling ensures physiologically realistic parameter values. But this also introduces a conceptual issue: if you're tuning parameters to match experimental outputs, then comparing your outputs to those same experiments isn't really independent validation. The authors should acknowledge this limitation. True validation would either need independent measurements not used for parameter fitting, or testing whether the model can predict results under new, untested conditions.

Reply: We agree with the reviewer that model validation should be performed on independent datasets not used for model development. To our best knowledge, current experimental techniques are insufficient to measure the neuromuscular system's input-output behavior at single-cell resolution. Due to this limitation, this study evaluated whether the pNMS can capture the key properties of the neuromuscular system that have been experimentally characterized at the system level, such as EMG–force relations (Figs 11 and 12). Further experimental studies are needed to validate the pNMS software using cellular-level input-output datasets for the neuromuscular system during voluntary contractions.

To address this issue, we have added a comparison with experimental findings (Line 991-1006) and clearly stated the limitations of the current PNMS software (Line 1060-1066) in the Discussion of the revised manuscript.

Reviewer #2:

General Comments
This was an ambitious, detailed effort to model most of the features influencing force and EMG production in mammalian muscle. Various conductances in both somatic and dendritic compartments of motoneurons were included in the model. Likewise, a large number of biophysical factors that shape motor unit force were included. These enabled simulations not only of isometric muscle force but forces arising while muscle changes length. Furthermore, the author provides some methods to handle the practical challenges of dealing with so many parameters. Indeed, this latter aspect seemed to be the main emphasis of the work. As such, this seems more a technical than scientific effort. Validation of the model output to previously published experimental work was meager and not that convincing. Furthermore, there were numerous minor concerns with the paper (detailed below).

Reply: We appreciate the reviewer for critical and constructive comments and recommendations on the manuscript.

Major Concerns
1. Throughout the manuscript, the author emphasizes that the outcomes using the present system match that developed on other platforms (e.g. Abstract "We validate the methodology using pNMS by comparing the simulation results with those from different software environments"). While this is important technically, it is of little scientific consequence. It is recommended that this matter be greatly downplayed.

Reply: I appreciate the reviewer bringing an important issue to our attention. Following the reviewer's recommendation, we removed the sentences from the Abstract (Line 36-38), Author Summary (Line 58-59) and Discussion (Line 797) in the revised manuscript.

2. While the EMG-force relations depicted in Fig. 11 F are roughly linear, they exhibit certain unphysiological features. In particular, they show an initial steep rise in EMG at low levels of force. This is opposite to what is found in some human muscles or otherwise the relation is strictly linear.

Reply: We apologize for the confusion caused by our mistake. The initial steep rise in the EMG-force relationship was due to the inclusion of the delay in force development at the initial phase of synaptic inputs to the motoneurons (see the below figure).

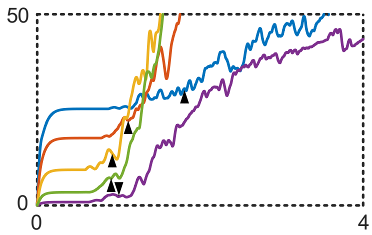

The inset of Fig. 11 E. Black arrowheads indicate the delay in force development during synaptic inputs to motoneurons.

This artefact was removed by considering the EMG and force data obtained after the delay of force development, as conducted in the previous study (J Neurophysiol. doi: 10.1152/jn.1993.70.6.2470.). Consequently, the initial steep increase was no longer evident in the revised EMG-force relationship. Furthermore, the MU population models comprising a mix of low and high threshold motoneurons tended to exhibit the linear relationship between EMG and force signal (yellow line in Fig. 11). In contrast, the MU population models comprising mostly low (purple line in Fig. 11) or high (blue line in Fig. 11) threshold motoneurons tended to exhibit the nonlinear EMG–force relationship. This result was in line with a previous study showing the variability in the EMG-force relationship between different muscles in humans (J Appl Physiol Respir Environ Exerc Physiol. doi: 10.1152/jappl.1983.54.6.1653.).

For clarification, we have included the sentence to explain the method for the construction of the EMG-force relationship in the Results (Line 744-746) and updated Fig. 11 accordingly in the revised manuscript.

3. The statement that “we reproduced the EMG–force relationships observed in cat” is without citation. Furthermore, it is difficult to imagine how one would obtain such an EMG-isometric force relation in the cat. The references to the work of Solomonow and colleagues is not really relevant in this regard because they used electrical stimulation. Such stimulation causes all active motor units to discharge in synchrony. Such synchrony minimizes cancellation of partially overlapping motor unit potentials (and reduction of EMG signal at high intensities) that occurs naturally (e.g. Keena et al. Amplitude cancellation reduces the size of motor unit potentials averaged from the surface EMG. J Appl Physiol 2006).

Reply: We agree with the reviewer that the natural (asynchronous and noisy) and experimental (synchronous and noise-free) excitation of muscle fibers may produce different EMG signals. For clarification, we have removed the references to cat studies for the simulation results shown in Fig. 11 in the Results of the revised manuscript (Line 993-1003).

4. Regardless, the Solomonow paper (EMG-force relations of a single skeletal muscle acting across a joint: Dependence on joint angle. J Electromyogr Kinesiol 1991) also showed a slow rise in EMG with increased force at low contraction intensities – opposite to what was shown in the present simulations. As a consequence, the degree to which the population simulations were validated by experimental data is not strong.

Reply: We have conducted an additional analysis to test whether the pNMS can capture the features of EMG-force relations observed in the cat experiment referenced by the reviewer. In that cat study, a stimulation protocol was applied to the soleus nerve, inducing the orderly recruitment of motor units from slow to fast types, with a progressive increase in firing rates from 10 to 60 Hz at various muscle lengths under anesthesia without afferent feedback. To mimic this condition, the population model involving the largest proportion of high-R_N MUs (purple line in Fig. 11 B) was simulated under four isometric conditions at physiologically maximal (0 mm), intermediate (-8 mm), minimal (-16 mm), and further shortened (-24 mm) MT lengths. We injected a triangular current input (i.e., a peak of 40 nA at 5 sec over 10

sec duration) intracellularly at the somata of motoneurons with passive dendrites for muscle contractions under full anesthesia without afferent feedback inputs. The peak of the current stimulation was chosen to ensure the recruitment of all motor units during the ascending phase of the triangular current injection. As a result, the time courses of the firing rate and EMG signal did not differ across muscle lengths (Fig. 12 A and B). However, the time course of force production depended on muscle length, reflecting the length-tension properties (Fig. 12 C). Consequently, the EMG–force relationship tended to be linear at the shortened length, but it became nonlinear as the muscle length increased (Fig. 12 D) as found in the cat experiment (Fig. 7 in the Solomonow paper). Notably, the iEMG–force relations were obtained by accounting for the delay (i.e., 0.5 sec) in force development at the initial phase of current injection to the motoneurons. These results support experimental finding that the muscle length may alter the EMG–force relationship.

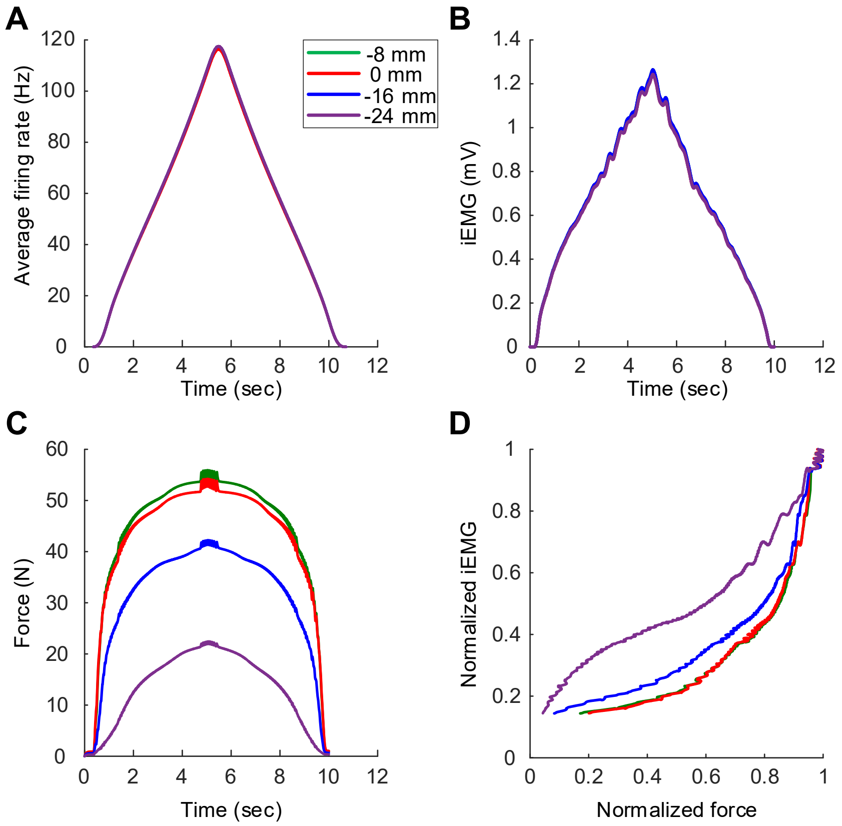

Figure 12 Length-dependent EMG–force relationship.

To clarify this issue, we have updated Fig. 12 and added the sentences regarding this additional analysis to the Results section of the revised manuscript (Line 764-780).

<u>Minor Concerns</u>

1. Please embed figures and table in the text of the manuscript. Scrolling back and forth to the end of an 86 page manuscript is tedious for reviewers.

Reply: We have embedded figures and tables within the text in the revised manuscript.

2. Why the focus on spinal motoneurons? Likewise, why restrict the approach to cat hindlimb muscles (Abstract "predict the population behaviors of neuromuscular cells in cat hindlimb muscles"). The utility of a modeling framework, such as advanced by the author, would be to use it flexibly to interrogate a wide range of motor unit populations, including those innervated by brainstem motoneurons and muscles throughout the body of various species.

Reply: The reviewer is correct. Our approach is not limited to spinal motoneurons and skeletal muscles. We intended to demonstrate how to use the modeling framework practically. For this purpose, we selected populations of neuromuscular cells experimentally characterized in adult cats. The current pNMS software offers two modes for flexible application programming. Users can build a neuromuscular system based on cat experiments in default mode and further modify cell properties in user-defined mode for broader applications.

For clarification, we have updated the Title, Abstract (Line 36-38), Author Summary (Line 58-59), Introduction (Lines 64, 74, 82, 90, 93, and 99), and Method (Lines 168, 288, and 310), Discussion (Lines 915, 1071, 1081, and 1099) in the revised manuscript.

3. (ln 130) "an inverse modeling framework has recently been proposed to overcome these issues by inferring and constraining effective model parameters directly from essential cell properties measured under various experimental conditions". I would take some exception to "recently" as this seems to have been the modeling approach taken in biology for some time.

Reply: For clarification, we have taken the term of "recently" out from the revised manuscript (Line 128)

4. Why is Dpath greatest for neurons with greatest input resistance (e.g. Fig 5B). High input resistance cells would likely be those that are physically smaller, and as such, would seem to be expected to have a shorter Dpath.

Reply: We apologize for the confusion that resulted from miscommunication on our part. The location of dendritic persistent inward current (PIC) across the motoneuron pool is a topic of debate. One study considering cell morphology (i.e., dendritic structure) with identical membrane properties has proposed that PIC location might be closer to the soma in smaller than larger motoneurons (J Neurophysiol 2007 doi: 10.1152/jn.00044.2007). However, another study, which considers both cell morphology and membrane properties, has shown the opposite result (J Appl Physiol 2017 doi: 10.1152/japplphysiol.00034.2017). We adopted the distribution of PIC locations (parameterized with D_{path}) from the second study, which better matched experimental observations from cats, such as voltage thresholds for PIC activation, their hysteresis, and input-output gains before full PIC activation.

For clarification, we have added the sentences to explain the selection of D_{path} distribution across the motoneuron population in the revised manuscript (Line 463-466).

5. Likewise, what is the physiological basis for the greater dendritic capacitance in the high input resistance cells (Fig. 5C5)?

Reply: In this study, the five passive membrane properties (i.e., $G_{m,S}$, $G_{m,D}$, G_C, $C_{m,S}$, and $C_{m,D}$) in were analytically determined to capture the five passive cell properties (i.e., R_N, τm, VA_{SD}^{DC}, VA_{DS}^{DC}, and VA_{SD}^{AC}) within the two-compartment modeling framework. Thus, the values of the passive membrane parameters do not necessarily reflect the values determined from the fully reconstructed models.

To alert readers to this critical issue, we have added the relevant sentences to the revised manuscript (Line 469-470).

6. What accounts for the abrupt drop in Fs (free calcium in soma, and linked to AHP duration) for neurons with input resistances > 1 Mohm? (Fig 5D1)

Reply: The abrupt drop in Fs in Figure 5 reflects the difference in the half AHP duration between the slow- and fast-type motoneurons. This phenomenon has been observed in MG motoneurons in cats (J Neurophysiol 1985 doi: 10.1152/jn.1985.53.5.1323). For clarification, we have added the sentences to the text in the revised manuscript (Line 470-472).

7. What is the intuition behind the inverted U shape in the relation between input resistance and fast sodium conductance (Fig. 5D2)?

Reply: We appreciate the reviewer's comment. The motoneuron models were built to match the experimentally characterized input resistance (R_N)-rheobase current (I_{rheo}) relationship (J Neurophysiol 1985 doi: 10.1152/jn.1985.53.5.1323) by adjusting peak conductance of fast sodium current (i.e., $G_{Naf,S}$) at the somatic compartment with passive dendrites. Motoneurons with low R_N tend to show high I_{rheo}, whereas motoneurons with high R_N need low I_{rheo} to initiate an action potential. However, motoneurons with low R_N and low I_{rheo} need more depolarizing inward current (i.e., $G_{Naf,S}$) to raise the membrane potential toward the threshold for an action potential.

For clarification, we have clearly addressed this issue in the revised manuscript (Line 472-473).

8. Fig. 5A description "Cell organization" is too general and uninformative. Indeed, "cell organization" could apply to the entire Fig. 5.

Reply: The reviewer is correct. We have replaced the phrase of "Cell organization" with "Distribution of cell indictor" in the revised manuscript (Line 480).

9. Related to Figs. 5E2 and E3, there is no need to make a plot when all cells were assigned the same values. Just simply state that in the text.

Reply: Following the reviewer's suggestion, we have removed Figs. 5 E2 and E3 and explained them in the revised manuscript (Line 476-477).

10. Why in Fig 1 is a multi-channel intramuscular electrode array depicted? The simulation have only to do with one channel. How would the present framework simulate the same intramuscular activity but from different electrode perspectives?

Reply: The reviewer is correct. The intramuscular electrode was incorrectly depicted in Figure 1B. In this study, average MUAP was simulated from the perspective of a single-channel intramuscular electrode in the middle of a motor unit territory. For clarification, we have updated Figure 1B in the revised manuscript.

11. (ln 376) “A step input with alternating brief excitatory and inhibitory pulses was applied to show the steady-state transition between two stable states” This is not clear. How can alternating depolarizing/hyperpolarizing pulses be used to reveal steady state behavior? What specifically do you mean by “brief”?

Reply: We apologize for this confusion. For clarification, we have revised the sentence in the revised manuscript (Line 439-442).
“A long-lasting step input with a brief (i.e., 50 ms) excitatory pulse followed by an inhibitory pulse with some delay (i.e., 10 sec) was applied to show the steady-state transition between two stable states, such as quiescent and regular firing or regular firing at different frequencies.”

12. (ln 429) “Each MT population model could replicate the force production that was previously experimentally shown for the variations in the stimulation frequency and MT length”. Please provide citation to support this statement.

Reply: We have provided the reference in the revised manuscript (Line 528).

13. What accounts for the non-physiological behavior of motor unit 10 in Figure 8, namely the sustained force after stimulation has ceased? Likewise what account for the non-physiological initial small rise in twitches and tetany before the steep rise in force as the outset of stimulation?

Reply: We appreciate the reviewer’s comment. In Figure 8, motor unit 10 illustrates the force production of cat gastrocnemius muscles. For these muscles, it has been experimentally observed that the force does not completely return to the baseline after stimulation ends (PLoS Comput Biol. doi: 10.1371/journal.pcbi.1011178). This phenomenon was reflected in the current version of the muscle model by modulating the threshold (i.e., C1i) and slope (i.e., C2i) of the calcium-activation curve in Module 2 (Figure 1 B). In addition, this difference in the calcium-activation curve in the fast-type muscle model gave rise to the initial slight rise before the onset of simulation. In the current pNMS version, C1i and C2i are the range model parameters, allowing the range of C1i and C2i to vary depending on the simulation purpose.

To clarify this issue, we have added the sentences (Line 553-557) in the revised manuscript.

14. What accounts for the non-physiological distributed EMG response to single stimuli in Fig. 8? Experimentally, such distributed responses are not seen even when stimulating the nerve quite distant from muscle.

Reply: The reviewer brings up an important point. We intended to clearly show the orderly delayed onset and offset of force production and the corresponding changes in EMG signal shape across a heterogeneous composition of MTs by applying a wide range (10-100 m/s) of axonal conduction velocity (CV). To clarify this issue, however, we have reset the CV distribution to 56-117 m/s to reflect the empirical data from a previous cat study (J Neurophysiol doi: 10.1152/jn.1987.57.2.510). Accordingly, we have updated the reference (Line 292), the sentence (557-559), and Figs. 7, 8, 9, 10, 11, and 12 in the revised manuscript.

15. Related to Figs. 8I and J, those are not “locomotor-like” movements. Locomotor movements are rhythmical and relatively brief. It would be helpful if the figures included what the input changes in length were associated with the force responses.

Reply: Following the reviewer’s suggestion, we have added the length variation associated with the force responses in Figs. 8I and J in the revised Fig. 8.

16. Fig. 9 seems to replicate most of what was already shown in Figs. 5 and 7. Therefore, there is no need to reshow those panels. Just mention what is different for the simulations of Fig. 10 from the parameters shown in Figs. 5 and 7. Also, if a parameter is not varied over the population, there is no need for a plot.

Reply: In Fig. 9, only three figures (D1, D2, and E2) are identical when compared to Figs. 5 and 7. We think it would be better to show the profiles of all range model parameters set across the population. For clarification, however, we have added a sentence to explain which parameters are not changed in the simulations shown in Fig. 10 in the revised manuscript (Line 628-630).

17. (lns 507-513). Please provide some physiological justification for the synaptic input schemes used. Also, define what is meant by “push-pull”.

Reply: Spinal motoneurons have been shown to receive both excitatory and inhibitory synaptic inputs from surrounding neurons. Three inhibition schemes were chosen to reflect background, recurrent, and reciprocal inhibitory synaptic inputs that have been experimentally observed. The term “push-pull” was used to describe an inhibition scheme in which inhibition decreases as excitation increases or vice versa via reciprocal inhibition. For clarification, we have added references and sentences to the revised manuscript (Lines 645-647 and 660-661).

18. (lns 516-517). Define the terms “onion skin” and “reverse firing”.

Reply: The terms “onion skin” and “reverse firing” were used to describe two types of firing patterns of motoneurons during ramp force production. The onion skin pattern indicates that the firing rate of low-threshold motoneurons is higher than that of high-threshold motoneurons. In contrast, the reverse pattern represents the opposite of the onion skin pattern, in which the firing rate of low-threshold motoneurons is lower than that of high-threshold motoneurons after firing acceleration on the ascending phase. For clarification, we have clearly described the terms “onion skin” and “reverse firing” in the revised manuscript (Line 691-696).

19. (ln 523). “the total intracellular electromyography signals” This should be intramuscular, not intracellular.

Reply: We have replaced the term of “intracellular” to “intramuscular” in the revised manuscript (Line 701).

20. (ln 537) “τ1–P 0.5relationship was updated to an exponentially decaying relationship derived from cat experiments”. Cite reference to justify this statement.

Reply: We have updated the sentence and added the references in the revised manuscript (Line 715-717).

21. (ln 543-545) “MU population models were simulated under the same push–pull synaptic inputs for MNs and isometric conditions for MTs as in Figure 10 D”. Why this form of input? What is the physiological justification, if any?

Reply: To our knowledge, synaptic inputs over the motoneuron pool have not been experimentally measured during voluntary muscle contraction. Thus, we chose the push-pull scheme based on a computational study suggesting that it better fits the human data compared to the constant and balanced inhibition schemes. We have added a sentence (Line 723-725) and reference (Elife 2024 doi: 10.7554/eLife.90624) in the revised manuscript.

22. There was no comment as to how reasonable the peak firing rates in the simulations shown in Fig. 10C were in comparison to recordings of cat hindlimb motor units. For example, Hoffer et al. (Cat hindlimb motoneurons during locomotion. II. Normal activity patterns. J Neurophysiol, 1987) rarely found firing rates in excess of 40 spikes/s across different speeds of treadmill locomotion.

Reply: We appreciate the reviewer bringing this important issue to our attention. To the best of our knowledge, there has been little experimental data on the relations of firing output and synaptic input across the motoneuron pool. Thus, this study used computational data from an anatomically reconstructed FR-type motoneuron model with a realistic distribution of synaptic inputs (J Physiol. 2005 doi: 10.1113/jphysiol.2005.099119). The previous research suggested that synaptic excitation of 450% of full Ia afferent inputs corresponded to an effective current of 20 nA injected at the soma and lead to the firing of FR-type motoneurons with active dendrites at up to 70 Hz

In this study, we applied the same effective synaptic current (20 nA) to the peak of the triangular current injection at the MN somata in Fig. 10 A. However, in Fig. 10 B, C, and D, the peak excitatory synaptic conductance (0.12 mS/cm^2) corresponding to the effective synaptic current (20 nA) was extended up to 0.3 mS/cm^2 to ensure all motor units are recruited during the ascending stimulation phase.

As a result, our corresponding FR-type motoneuron model fired at a peak rate of 65 Hz with current injection at the soma and 48 Hz under noise-free synaptic input to the dendrite (Figs. S1 and S2 in Supplemental Information 1). In addition, our

motoneuron population matched the systematic variation in voltage thresholds for PIC onset and two firing output-input intensity (F-I) gains before full PIC activation (i.e., F-I_N for synaptic input and F-I_S for current injection) across the motoneuron pool in cat hindlimb muscles, as shown in our previous study (J Appl Physiol 2017 doi: 10.1152/japplphysiol.00034.2017).

Furthermore, it remains unclear what and how synaptic inputs are organized and activated for motoneurons during locomotion. The low firing rate (< 40 Hz) observed during cat walking is likely due to the combinatory effects of excitatory, inhibitory, and neuromodulatory inputs from various sources (Physiol Rev 2000 doi: 10.1152/physrev.2000.80.2.767). In this context, the pNMS may serve as a flexible framework for systematically examining the impact of various intrinsic and extrinsic factors on the firing patterns of the motoneuron pool during locomotion.

To clarify this issue, we provided sentences explaining the stimulation protocol and firing behavior in the Results (Lines 661-663) and the Discussion sections (Line 948-964) of the revised manuscript.

23. The responses in the different motor units shown in Fig. 10 are confusing and unclear. Part of the problem is the use of the same color traces for different motor units. For example, there seems to be no force produced by motor unit 1 in panel A3. In panel B3, there appears to be a blue line that stays at zero force throughout. If that is MU 2, then why does it not get recruited. If it is MU 8, why does it not get recruited even though MU 10 appears to be activated. What accounts for the sustained activity in MUs 9 & 10 (panels B1, C1, D1) but not in other units?

Reply: We feel sorry for this confusion. The blue lines near zero force throughout in panel A3 and B3 indicated MU 1. In addition, the sustained activity in MU 9 and 10 indicated the sustained PIC in high threshold motoneurons when they were recruited by synaptic inputs at their dendrites. To bring clarity, we replotted Figs. 6, 8 and 10 with different colors for different MTs and MUs in the revised manuscript.

24. In Fig. 10, panels A7, B7, C7, and D7 are unnecessary.

Reply: Following the reviewer's suggestion, panels A7, B7, C7, and D7 were removed from Fig. 10 in the revised manuscript.

25. (lns 569-572) "0.0064 mS/cm 2 at the intermediate MT length (i.e., −8 mm), and 0.0128 mS/cm 2 at the lengthened MT length (i.e., 0 mm) under isometric conditions, as reported in a previous study." Please provide citation that supports these afference conductances.

Reply: This sentence has been removed from the text. The reason was that we have reanalyzed the influence of muscle length on EMG-force relations without Ia afferent feedback in the revised manuscript (Line 764-780). Please refer to our reply to the fourth reviewer's major concern.

26. Legend to Figure 11. Change throughout "individual motor unit populations" to "different motor unit populations"

Reply: Changed in the revised manuscript (Line 757-761).

27. (ln 1036) “motor unit in human hindlimb muscles” should be “human leg muscles”

Reply: Updated in the revised manuscript (Line 254 in Appendix A in Supplemental Information 5).

28. (lns 1052 – 1057) There are a large number of constants included in these equations without any explanation as to how they were determined. Likewise for ln 1079.

Reply: The equations and constants were fully addressed in our previous study. We have explained them briefly along with the citation in the revised manuscript (Lines 276-277, 299-300, 308-309, and 318-319 in Appendix B in Supplemental Information 5).

29. (ln 1772) “The lower panel shows a cross-section of the muscle–tendon complex” Should be longitudinal, not cross – section.

Reply: Corrected in the revised manuscript (Line 173).

30. Each table in the Methods should have a number and a title

Reply: Updated in the revised manuscript (Lines 305, 314, 324, and 346 in Appendix B in Supplemental Information 5).

31. For the table shown just after ln 1096, please indicate what parameter is represented in the table

Reply: $G_{Cal,D}$ has been presented as a function of R_N and D_{path} in the table. (Line 324 in Appendix B in Supplemental Information 5).

32. The table just after ln 1102 is not needed. Likewise for the table following line 1107.

Reply: Both tables have been removed from the revised manuscript (Line 326-331 in Appendix B in Supplemental Information 5).

33. Each of the columns in the table after ln 1121 needs a label.

Reply: Following the reviewer’s suggestion, the table has been updated in the revised manuscript (Line 346 in in Appendix B in Supplemental Information 5).

Reviewer #3:
This is an interesting manuscript that introduces pNMS, an open-source software tool designed to construct biophysical population of reduced models of motor units (MUs). The tool provides pre-packaged parameter sets to construct motor unit's population of heterogeneous properties, integrates a built-in simulation engine to solve the models ODEs, and supports parallel simulations. The author validated the tool results against general-purpose simulators such as NEURON and XPPAUTO. The work's key value lies in its pre-packaged MU models and the integration of motoneuron, muscle-tendon, and whole motor unit dynamics within the same environment parallelization and scalability add practical advantages. However, several aspects require some clarification, particularly regarding usability, comparisons with existing tools, and definitions such as forward vs. inverse modeling.

Reply: We appreciate the reviewer for critical and constructive comments and recommendations on the manuscript.

Specific comments are listed below.
1. The distinction between forward and inverse modeling frameworks is insufficiently explained in both the introduction and discussion. Both appear to use experimental data for parameter optimization, but how they differ in philosophy and application should be explicitly clarified. It is my understanding that some properties like the ion channel kinetics can be optimized once and then used with different conductance for different models - would that be considered forward or inverse modeling? Would framework such as Caillet et al. (2023) qualify as inverse modeling?

Reply: We apologize for the confusion that resulted from a miscommunication on our part. In this study, the terms 'forward' and 'inverse' modeling were used to differentiate the methods of determining parameter values. Forward modeling involves numerically optimizing multiple model parameters to reproduce a set of target cell outputs in response to representative stimulation protocols. In contrast, inverse modeling infers and constrains effective model parameters directly from essential cell properties measured under various experimental conditions rather than cell outputs. For instance, the model parameters (i.e., $G_{m,S}$, $G_{m,D}$, G_C, $C_{m,S}$, and $C_{m,D}$) of the passive membrane properties were determined by the inverse equations for the cable properties (i.e., R_N, τm, VA_{SD}^{DC}, VA_{DS}^{DC}, and VA_{SD}^{AC}) in the reduced motoneuron model (see Appendix B for more details). In the current version of pNMS, all relevant model parameters were uniquely determined directly from the target cell properties (see Tables 1 and 2).

To clarify this issue, we clearly stated the definition of the two terms in the introduction of the revised manuscript (Lines 121-123 and 128-131).

2. The author should clarify whether pNMS provides flexibility for users to implement custom models or is mainly tailored to pre-defined configurations. If flexibility is provided, it may be helpful to discuss how (and perhaps why) the user would accomplish this. Likewise, it may be helpful to add a section dedicated to how the user would modify/customize the simulations settings. I understand that changing the parameters on the excel sheets is one way.

Reply: We appreciate the reviewer bringing this critical issue to our attention. The implementation of user-defined custom models is currently limited in the pNMS environment. This limitation is partly due to the use of an inverse modeling approach that requires the analytical determination of model parameter values directly from target cell properties to automate population model construction. However, the current pNMS version provides flexibility in modifying the distribution of cell properties and relevant model parameters across the population model (Tables 1 and 2). For homogeneous population models, the user can change model parameter values in the Excel spreadsheet (i.e., model parameter files in Supplemental Information 3). For heterogeneous population models, the distribution of cell properties can be defined by users using built-in functions or importing user-defined data files (see Appendix B in Supplementing Information 5). Likewise, the user can also update the distribution of individual range model parameters using built-in functions or importing user-defined data files (see Appendix B in Supplementing Information 5). Furthermore, users can specify initial values for model equations, simulation time and resolution, parallel environment configuration, model inputs, and simulation outputs to plot online using built-in functions or user-defined data files. All these modeling and simulation settings can be performed via a command-line application programming interface (API), making pNMS efficient and easy to integrate into broader applications (see Table 3 for API functions).

For clarification, we have added a section explaining how users can customize the simulation settings under the current version of pNMS in the Discussion of the revised manuscript (Lines 856-873).

3. The manuscript would greatly benefit from more explicit comparisons with existing tools. This includes comparisons with general-purpose simulators (NEURON, XPPAUTO, and Population/network-focused tools: NetPyNe) and MU focused models/tools (Cisi & Kohn (2008), Elias & Kohn (2013, 2014)). Perhaps this could be achieved by splitting discussion Section 2 (Reliability and Efficiency) into two sections 1) Validation and reliability, 2) Comparison to existing tools. Also please elaborating on what you mean by “the discrepancy in the computational models” in line 631.

Reply: Following the reviewer's suggestion, we have split Section 2 of the Discussion into 'Validation, reliability, and flexibility' and 'Comparison to existing tools'. In the later section, we have added the sentences describing comparisons with existing tools, including those mentioned by the reviewer.

Biophysical models for motoneurons have been simulated mostly using general-purpose software such as NEURON and XPPAUTO. In contrast, special-purpose software has been developed for muscle fibers and motor units. Typically, general-purpose software tools offer great flexibility in developing user-customized models, whereas special-purpose tools are limited to the canonical models embedded in their software. The current version of pNMS uses the canonical models for motoneurons and muscle fibers. However, unlike prior software tools, the canonical models are constrained directly by empirically measurable cell-specific properties. Furthermore, those models were designed and implemented as classes following the object-

oriented programming principle, making maintenance and code extension easy and efficient.

To our knowledge, all existing software tools use a forward modeling scheme in which model parameters are treated as free parameters. Thus, users must optimize the model parameters to reflect the experimental data. However, the current version of pNMS offers both forward and inverse modeling modes, allowing the user to choose whether model parameters are analytically determined directly from experimental data or numerically optimized.

General-purpose tools have provided both graphical user interfaces and command-line interfaces (or application programming interfaces). In contrast, the special-purpose tools have provided only graphical user interfaces for setting the model and simulation conditions. The current pNMS version offers only a command-line interface, which is known to be efficient for simulation management and flexible for integration in broader applications. Future studies would be needed to design and develop a graphical user interface for pNMS, improving user accessibility and software usability.

Some software tools enable parallel simulation through a high-performance computing system or a web-based environment. The current pNMS version offers a flexible parallel simulation configuration, allowing users to run simulations on a single multicore processor, a high-performance computer cluster, or a web-based environment. Furthermore, the pNMS provides automated construction of a parallel environment without additional programming or library installation.

For clarification, we have added a new section to the Discussion of the revised manuscript (Line 877-910).

4. On the first page of Methods, too many terms are introduced at once (CPs, RMPs, CMPs, cell classifier, MT peak force). The author may consider adding more context or examples for these terms first. Also, please clarify how parameters are categorized as CMPs vs. RMPs—is this a modeling convention, or is it based on inherent properties (e.g., are ion channel kinetics CMPs or RMPs)?

Reply: Following the reviewer’s suggestion, we have added examples for the terms of CPs, RMPs, CMPs, cell classifier, and MT peak force in the Methods section. In addition, the terms 'CMP' and 'RMP' were used to distinguish the model parameters that are subject to change to reflect the cell property. This annotation has been applied as a modeling convention rather than as an inherent property. To alert this issue to the readers, we have added the sentence to the Methods of the revised manuscript (Lines 197-199, 202-204, and 211-214).

5. The author my consider rewording lines 143-144 to “methodology that facilitates motor believe models developments and simulation” Instead of using “minimizes manual”. I believe manual tunning would be needed for any customization or new model.

Reply: Following the reviewer’s suggestion, we have updated the sentence in the

revised manuscript (Line 141-142).

6. The term “network folder” is unclear—please clarify whether this is standard terminology in parallel computing.

Reply: The reviewer is correct; the network folder is a standard terminology used in the field of parallel computing (Line 346).

7. The term “neuromuscular cells” is not the most accurate here. I think the author is focusing on motoneurons specifically, and not muscle cells. I suggest using motoneurons. Or perhaps “motor units’ models”.

Reply: Following the reviewer’s suggestion, we have changed “neuromuscular cells” to “motoneurons” in the revised manuscript (Line 134).

8. Line 91: The phrase “Computer modeling has recently been employed” is inaccurate. Modeling has a long history; the sentence would read better without “recently.”

Reply: We have deleted “recently” in the revised manuscript (Line 89).

9. Please clarify what you mean by using “mathematical tools” lines 98-99.

Reply: The phrase “mathematical tools” was used to indicate that well-established mathematical theories, such as nonlinear dynamical systems theory, can be readily applied to analyze the reduced models. To clarify this issue, we have provided an example for mathematical tools in the revised manuscript (Line 96-97).

10. It would improve readability to avoid abbreviations in the headers, tables and figures, this will make it more readable (MT, MN, MU, etc)

Reply: Following the reviewer’s suggestion, we have revised the headers, tables, and figures without abbreviations in the revised manuscript (Lines 223, 246, 251, 278, 283, 427, 479, 498, 506, 566, 575, 592, 634, 666, and 752).

11. The author may consider using less abbreviation if possible. Some terms like CIs, are only used very few times, so it would be better not to abbreviate terms used less frequently.

Reply: We apologize for the confusion. We used terms such as CP, RMP, CMP, and CI primarily to formulate the inverse modeling framework in a mathematical form. For this reason, we believe that using abbreviations would be more efficient for maintaining clarity and consistency throughout the manuscript.
